# Protection afforded by respirators when performing endotracheal intubation using a direct laryngoscope, GlideScope®, and i-gel® device: A randomized trial

**DOI:** 10.1371/journal.pone.0195745

**Published:** 2018-04-19

**Authors:** Hyunggoo Kang, Yoonje Lee, Sanghyun Lee, Yeongtak Song, Tae Ho Lim, Jaehoon Oh, Juncheol Lee, Hyungoo Shin

**Affiliations:** 1 Department of Emergency Medicine, College of Medicine, Hanyang University, Seoul, South Korea; 2 Convergence Technology Center for Disaster Preparedness, Hanyang University, Seoul, Republic of Korea; 3 Department of Emergency Medicine, College of Medicine, Hallym University, Seoul, South Korea; National Yang-Ming University, TAIWAN

## Abstract

Emergency physicians are at risk of infection during invasive procedures, and wearing a respirator can reduce this risk. The aim of this study was to determine whether the protection afforded by a respirator during intubation is affected by the type of airway device used. In this randomized crossover study, 26 emergency physicians underwent quantitative fit tests for a N95 respirator (cup-type or fold-type) before and during intubation with a direct laryngoscope, GlideScope®, or i-gel® airway device. The primary outcome was the fit factor value of the respirator and the secondary outcome was the level of acceptable protection provided (percentage of fit factor scores above 100). Compared with the GlideScope and i-gel device, the fit factor values and level of acceptable protection provided were lower when physicians wore the cup-type respirator while intubating using the direct laryngoscope (200 fit factor [152–200] and 200 fit factor [121.25–200] versus 166 fit factor [70–200], 100% and 100% versus 75%, respectively; all *P* < 0.001). There were no significant differences in the fit factor value or level of acceptable protection provided when the physicians wore the fold-type respirator while intubating using any of the three airway devices (all *P* > 0.05). The type of airway device used for endotracheal intubation may influence the protective performance of some types of respirators. Emergency physicians should consider the effects of airway device types on fit factor of N95 respirators, when they perform intubation at risk of infection.

## Introduction

Emergency physicians are the front-line health care workers at highest risk of exposure to airborne and aerosolized infectious hazards during invasive and emergency procedures, such as endotracheal intubation [1]. Various devices and guidelines have been developed and used clinically to minimize the risk of exposure. For example, many health care organizations recommend use of an N95 filtering facepiece respirator [2–4]. The National Institute for Occupational Safety and Health also ranks the protective performance of the various N95 respirators according to their filtering efficiency [5,6]. Unfortunately, emergency physicians can still be exposed to infectious hazards while wearing certified respirators if the respirator is not correctly fitted to the wearer’s face [7]. One study revealed that inward leakage of contaminants because of an incomplete face seal was 3–5-fold greater than that via penetration of the filter itself [8]. Furthermore, movement of the head can affect the fit of the respirator if there is a change in the relative positions of the face and the respirator, which can potentially cause leaks in the face seal [9,10]. A direct laryngoscope is used as a first-line airway device during endotracheal intubation [11,12]. Emergency physicians must approach the patient’s mouth and manipulate the airway structures to expose the vocal cords. The risk of infection can increase during endotracheal intubation using a direct laryngoscope as a result of aerosolized hazards generated during coughing and movement during intubation [13–15]. Movement during endotracheal intubation using a direct laryngoscope has been reported to influence the protective performance of respirators [16]. The GlideScope® (GVL; Verathon, Bothell, WA, USA) has a hyperangulated blade, the tip of which is equipped with a camera. The i-gel® (IGEL; Intersurgical Ltd, Wokingham, UK) is a single-use supraglottic airway device comprising a soft gel-like cuffless mask, a narrow-bore gastric drain tube, and an integral bite block. No movement is needed to expose the vocal cords when intubations are performed using the GVL or IGEL. To the best of our knowledge, there are no reports on the effect of movement during endotracheal intubation using the various types of airway devices on the protective performance of respirators. The aim of this study was to determine whether different types of airway devices affect the protection afforded by a respirator during endotracheal intubation.

## Materials and methods

### Study design

This randomized crossover study was performed at a Korean university medical center in September 2016. The local ethics committee approved the study protocol in January 2016 (Hanyang university seoul hospital institutional review board: HYUH 2015-11-019-004). All the physicians who participated in the study provided informed consent. No patients were enrolled in the study. The study protocol was registered with the Clinical Research Information Service (cris.nih.go.kr: KCT0001803) before the study was initiated. This study had been designed and performed according to the consort checklist of information to include when reporting a randomized trial assessing non-pharmacologic treatments (NPTs).

### Participants

We recruited healthy emergency physicians up to 60 years of age working at the same tertiary medical center in August 2016, all of whom had performed at least 50 intubation procedures using a direct laryngoscope. Subjects were excluded if they had lung disease, high blood pressure (i.e., systolic >160 mmHg, diastolic >95 mmHg), a wrist disorder, or low back pain. All participants in the study signed a written consent form before inclusion. The sample size was calculated based on a pilot study of differences in the fit factor values between two N95 respirators during intubation using three different types of airway device. In that study, the mean (± standard deviation) fit factor values were 128.71 ± 41.07 for the Macintosh laryngoscope, 170.27 ± 39.25 for the GVL, and 161.46 ± 38.43 for the IGEL during intubations. The sample size was calculated using G-power 3.1.2® (Heine Heinrich University, Düsseldorf, Germany). Sample size had been calculated based on the assumption of use F tests ANOVA: Repeated measures, between factors. An alpha error was 0.05, a power was 0.8 and effect size f was 0.4515279. While allowing for a 10% dropout rate, we estimated that 26 subjects would be needed.

### Equipment and materials

**Fig 1:**
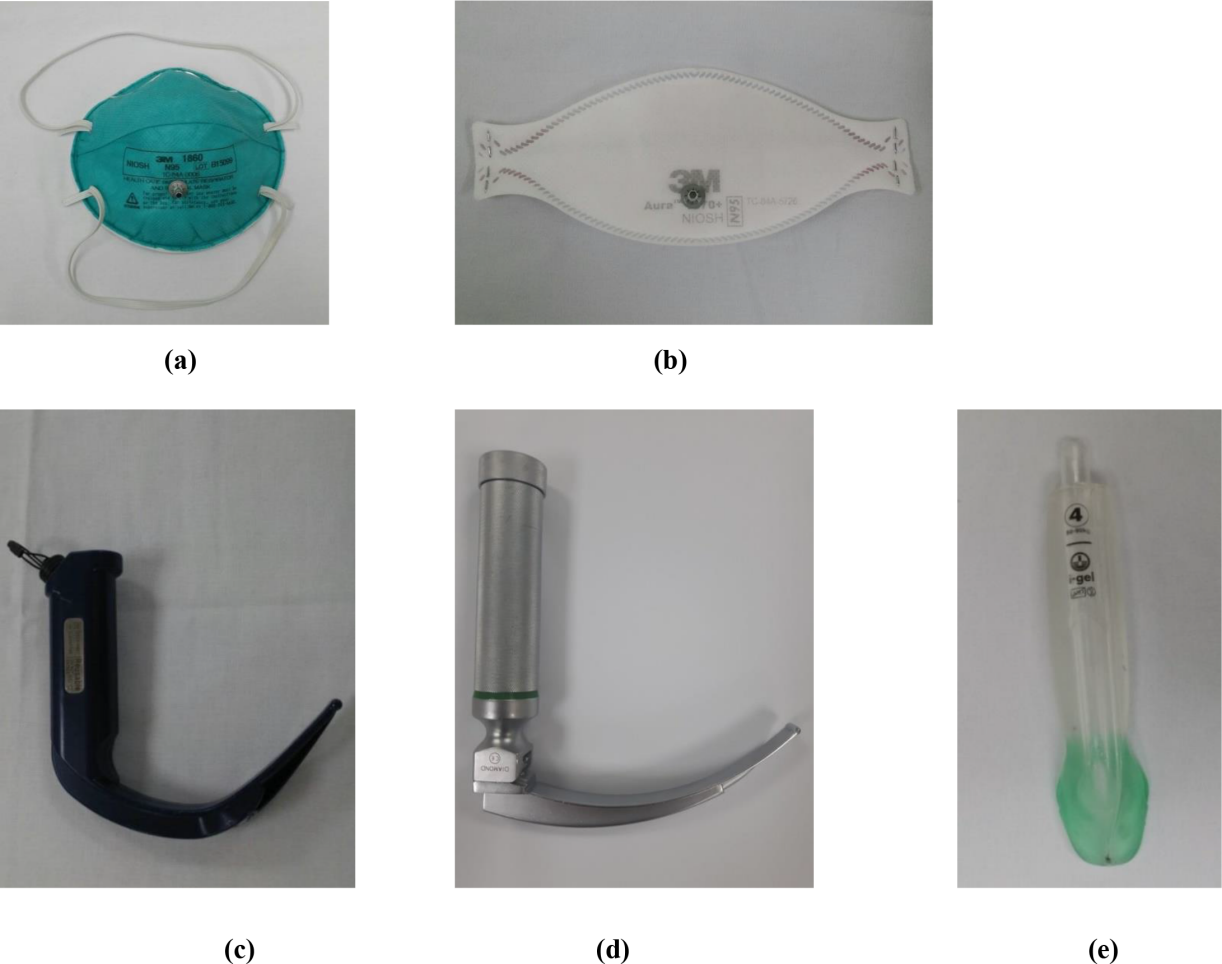
The quantitative fit test was performed using the Porta-Count Plus. (a) Cup-type respirator, which is preformed to a cup shape. (b) Fold-type respirator, which is flexible and free-folded. (c) Macintosh laryngoscope, which has a size 4 curved blade with a Satin Slip stylet. (d) GlideScope® with a GlideRite rigid stylet. (e) I-gel® airway device.

Two types of respirator, both with an N95 filter, were selected for this study, i.e., a cup-type respirator, which is pre-formed into a cup shape (1860 or 1860S; 3M, Elyria, OH, USA) and a fold-type respirator, which is flexible and free-folded (1870; 3M). These two respirators are supplied by a leading manufacturer and were used in emergency medical centers during the Middle East respiratory syndrome epidemic in Korea in 2015. The quantitative fit test for the N95 respirators was performed using the PortaCount® Plus (TSI Inc, St. Paul, MN, USA; Fig 1). This device is equipped with two sampling tubes. One sampling tube is exposed to the atmosphere to measure the ambient particle concentration and the other is connected to the respirator to measure the particles therein. The other end of each sampling tube is connected to the PortaCount Plus. The fit factor value is calculated as the ratio of the concentration of particles in the air outside the respirator to the concentration of particles that have leaked into the respirator. The fit factor has a maximum value of 200, and a value >100 indicates that the respirator has passed a quantitative fit test [17]. The weight of the tube is supported by a wire placed around the operator’s neck.

Intubation was performed using the Laerdal airway management trainer (Laerdal Medical, Stavanger, Norway) and the following three types of airway devices: a Macintosh laryngoscope, which has a size 4 curved blade with a Satin Slip intubating stylet (Mallinckrodt Medical, St. Louis, MO, USA); a GVL with a GlideRite rigid stylet; and an IGEL. An endotracheal tube with a standard cuff and an internal diameter of 8.0 mm (Lo-Contour Murphy; Mallinckrodt Medical, Athlone, Ireland) was used. When using the Macintosh laryngoscope, the manikin’s head and neck were placed in a sniffing position using several rolled sheets as well as in a neutral position by placement on a bed (transport stretcher, 760 mm × 2110 mm, 228 kg, Stryker Corporation, Kalamazoo, MI, USA). The height of the bed was adjusted to approximately that of the physician’s mid-chest level.

### Interventions

**Fig 2:**
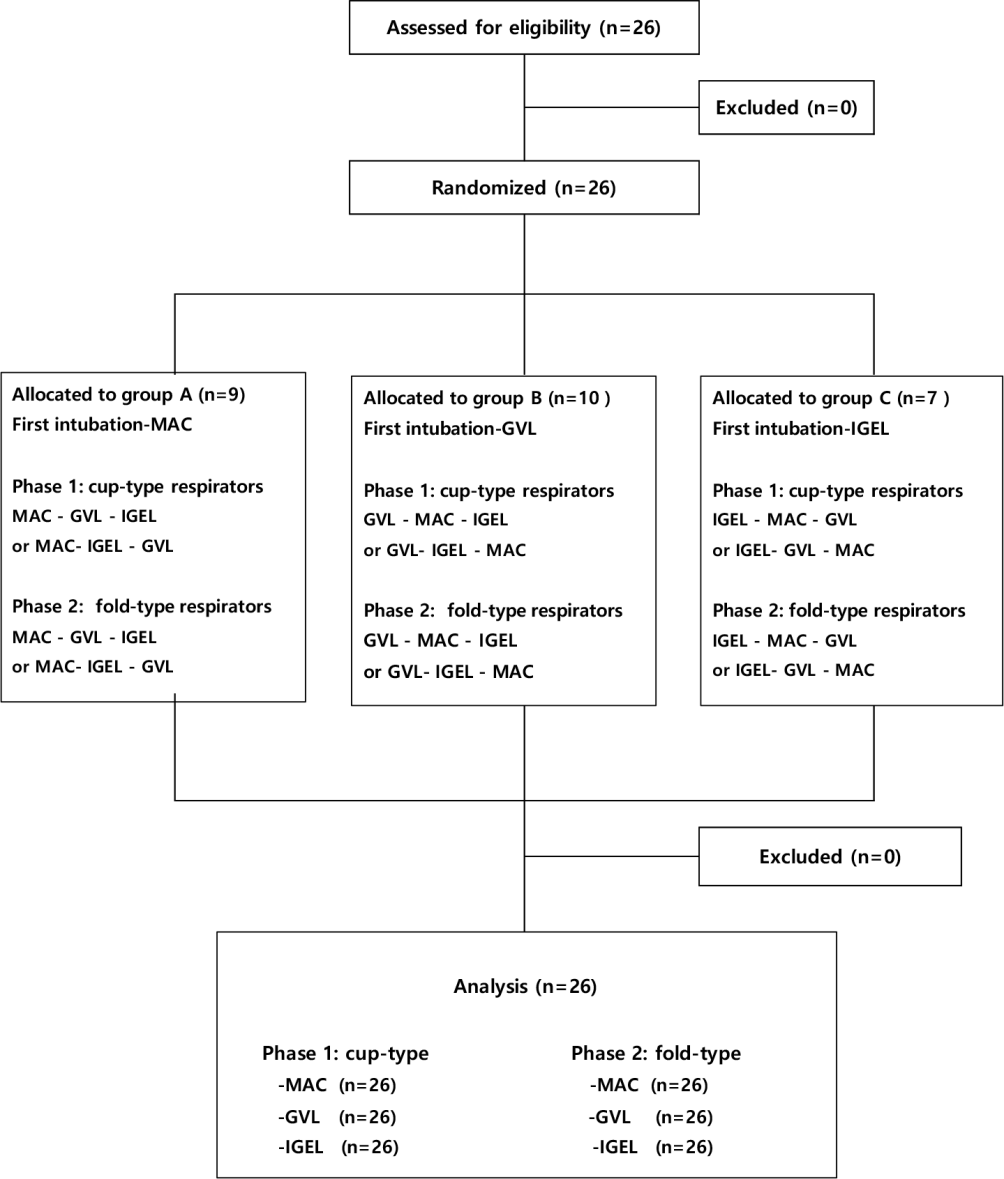
Diagram showing the flow of participants through the study.

All participants completed a brief questionnaire for collection of demographic data (age, sex, body weight, and height) and previous clinical experience of wearing an N95 respirator while performing intubations using the three types of airway device. Twenty-six physicians were enrolled and randomly allocated to one of three groups according to the type of airway device used first and second (www.random.org; Fig 2). All study participants were required to refrain from smoking, eating, chewing gum, and drinking (except for still water) for at least 30 minutes before starting the quantitative fit test. This simulation trial was performed in a triage room (24.3 m^3^) without air conditioning in one emergency medical center. We used a particle generator (TSI, model 8026) to generate a sodium chloride aerosol to ensure that the ambient air contained at least 100 particles in the correct size range per milliliter. All study participants were assigned respirators based on their face and lip length measurements, as recommended by the Los Alamos National Laboratory [18]. Before starting the trials, the subjects were allowed to practice applying each type of N95 respirator and received brief education about the wearing of these respirators by reading the infection control manuals. Ten minutes before starting the trials, they were allowed to practice intubations with each of the three airway devices without wearing the respirator to familiarize themselves with the Laerdal airway management trainer. The fit factor value for each type of N95 respirator was measured in three simulation scenarios, i.e., using the Macintosh laryngoscope, the GVL, and the IGEL. Finally, each subject performed a series of 6 quantitative mask fit tests. Before applying the N95 respirator, one sampling tube was connected to a probe inserted into the respirator without deforming the shape of the respirator. An interval of 10 minutes was allowed before changing the type of simulation scenario and an interval of about 5 minutes was allowed before changing the type of N95 respirator used. A seal check was performed before each change of simulation scenario or respirator.

### Outcomes

The primary outcome was the fit factor value for each type of N95 respirator. The secondary outcomes were the percentage of fit factor values ≥100 (indicating that the respirator provided an acceptable level of protection) [17], preference for a particular type of N95 respirator, intubation time, and intubation success rate. The fit factor and intubation time were measured from the start point (when the physician inserted the blade between the teeth after being given the pre-recorded command to start) to the end point (the first manual ventilation after intubation, regardless of whether or not the manikin’s lungs were inflated). Intubation failure was deemed to have occurred when the tip of the tube was placed in the esophagus or oral cavity instead of the trachea or when the intubation time was 90 seconds or more [19,20]. The subjects were asked to choose the type of respirator they would prefer to wear when performing intubation using each type of airway device in a situation where there was a significant risk of infection.

### Statistical analysis

The data were entered into an Excel spreadsheet (Microsoft Corporation, Redmond, WA, USA) and analyzed using SPSS Statistics for Windows software, version 18.0 (IBM Corp., Armonk, NY, USA). The categorical data are shown as the frequency and percentage and the continuous data as the median and interquartile range because they were not normally distributed. The Friedman test was used for continuous variables when comparing the fit factor values for 3 types of airway devices when intubating wearing cup-type and fold-type N95 respirators. A post hoc analysis was conducted with the Wilcoxon signed-rank test using Bonferroni correction. Wilcoxon signed-rank test was used for continuous variables when comparing the fit factor values for cup-type and fold-type N95 respirators when intubating using each type of airway device. A *P*-value < 0.05 was considered to be statistically significant.

## Results

### Subject demographics and clinical experience

All 26 subjects completed the study, and their demographic characteristics and experience of performing endotracheal intubation are summarized in Table 1. Nineteen subjects were fitted with a model 1860 respirator and seven with a model 1860S respirator according to their face and lip measurements.

**Table 1:** Demographic characteristics and clinical experience.

Variable	Value
Male sex, n (%)	26 (100)
Age (years)	31 (28–35)
Height (cm)	177 (171–180)
Weight (kg)	75 (69–80)
Postgraduate experience (years)	4 (3–6)
Intubations performed (n)	80 (50–100)
Face width (mm)	138.17 (10.84)
Face length (mm)	119.78 (9.59)

Categorical variables are shown as the number (percentage). Continuous variables are shown as the median (interquartile range).

### Fit factor values during intubation using each type of airway device while wearing a cup-type respirator

**Fig 3:**
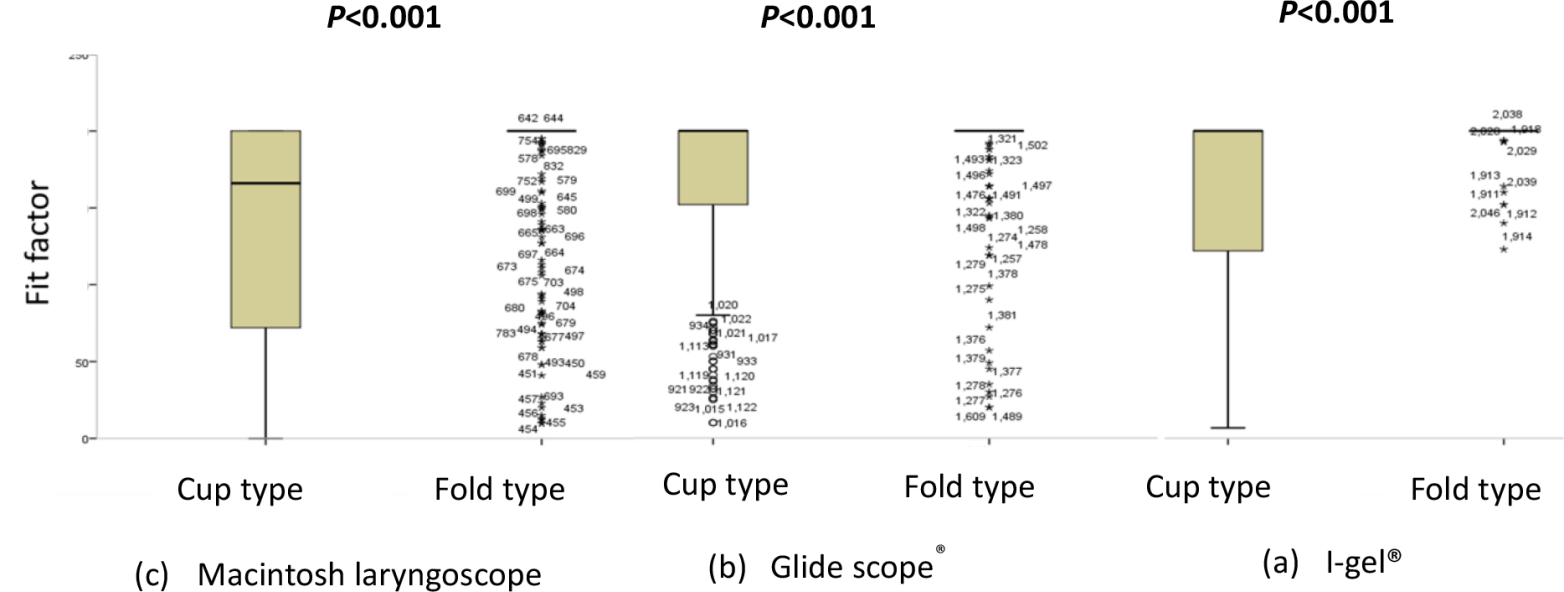
Fit factor values for the two types of N95 respirator when used for intubation using three types of airway device. The fit factor value for the valve type was decreased during intubation. There were significant differences between the fit factor values for the cup-type respirator and the fold-type respirator when intubations were performed using the three different airway devices. ∘Outlier value more than 1.5 times the upper or lower limit.

There was no significant difference in the fit factor value or protection afforded by the cup-type respirator when using the GVL or IGEL during intubation (*P* = 0.453 and *P* = 0.735, respectively). However, the fit factor value and protection afforded by the cup-type respirator when the Macintosh laryngoscope was used were lower than those of the other two airway devices (*P* < 0.05; Table 2). The median fit factor for the cup-type respirator was lower than that of the fold-type respirator in the Macintosh laryngoscope scenario (*P* < 0.001). There were no differences in the median fit factor values between the two types of N95 respirators in the GVL and IGEL scenarios, but the dispersions were significantly different between the two respirators (all *P* < 0.001; Fig 3).

**Table 2:** Fit factor values for N95 respirators in the baseline and intubation scenarios (n = 26).

				Type of N95 respirator				
		MAC	GVL	IGEL	*P*-value	MAC	MAC	GVL
		(n = 24)	(n = 24)	(n = 24)		vs GVL	vs IGEL	vs IGEL
Cup type	Fit factor	166	200	200	<0.001	<0.001	<0.001	0.453
		(70–200)	(152–200)	(121.25–200)				
	Adequate protection* (%)	75	100	100	<0.001	0.006	0.002	0.735
		(44–100)	(100–100)	(83.3–100)				
Fold type	Fit factor value	200	200	200	1	1	1	1
		(200–200)	(200–200)	(200–200)				
	Adequate protection*	100	100	100	0.01	0.176	0.028	0.068
	(%)	(90.4–100)	(100–100)	(100–100)				
Intubation time	Intubation time^†^	17.65 (5.67)	15.67 (7.54)	15.74 (5.63)				
	(seconds)							
Intubation success rate	Intubation success rate	26 (100)	26 (100)	26 (100)				
	(%)							
Preference	Preference	5 (19.2)	12 (46.2)	9 (34.6)				

Categorical variables are shown as the number (percentage). Continuous variables with a normal distribution are shown as the mean (standard deviation). Nonparametric variables are shown as the median (interquartile range). Cup-type respirator: 3M model 1860. Fold-type respirator: 3M model 1870.

*Adequate protection is the percentage of fit factor values ≥100.

^†^Intubation time, interval between inserting the blade into the mouth and the first ventilation.

MAC, Macintosh laryngoscope; GVL, GlideScope; IGEL, i-gel.

### Fit factor values during intubation using three different airway devices while wearing a fold-type respirator

There was no significant difference in the fit factor value for the fold-type respirator when performing intubations using the three airway devices (*P* = 1.000). The level of protection afforded by the fold-type respirator was at least 90% during intubation with all three airway devices (Table 2).

### Respirator preferences

Twelve subjects (46.2%) preferred the GVL, 9 (34.6%) preferred the IGEL, and 5 (19.2%) preferred the Macintosh laryngoscope for prevention of infection during intubation while wearing an N95 respirator.

### Intubation time and success rate

The intubation time was longer when using the Macintosh laryngoscope than when using the other airway devices. However, the intubation success rate was 100% for all three devices.

## Discussion

Respirators with an N95 filter are recommended to protect physicians against infection via droplets or aerosol during invasive procedures [2–4]. Movements during endotracheal intubation using a direct laryngoscope could influence the protective performance of some respirators [16]. However, there have been no studies of the effects of the different types of airway devices on the protection afforded by respirators during intubation in the emergency room.

Or et al. demonstrated that training undergraduate nursing students to fit respirators correctly increased the likelihood that the respirators would be effective [21]. However, a component analysis of respirator user training revealed that adequate knowledge alone did not ensure correct use of a respirator in clinical practice [22]. All subjects in the present study received real-time feedback after their respirator training based on the manufacturer’s manual. This type of training course could help to improve the fit for all types of respirators.

Our simulation study revealed that different movements performed during intubation could influence the protective performance of some respirators depending on the type of airway device used. Bending from the waist with movement of the head is required to expose and visualize the vocal cords during intubation using a direct laryngoscope, and we believe that these movements can decrease the protection afforded by some respirators [9,10]. However, intubations using a video laryngoscope or a supraglottic device do not require movement to expose and visualize the vocal cords directly. Movement is not necessary using a video laryngoscope because there is a camera on the tip of the blade and a display screen is used.

Moreover, when compared with direct laryngoscopy, use of a video laryngoscope could reduce the intubation time, which might also contribute to more protection and limit exposure to infection [23,24]. In the present study, the adequate protection and fit factor values differed according to the type of airway device scenario when intubations were performed wearing the cup-type respirator, but not when wearing the fold-type respirator (Table 2). These findings suggest that the different movements required when using the different airway devices can affect the protection provided by the different types of respirators. We believe that the user should take into account the movements required when using each type of device for intubation when selecting and fitting a respirator for procedures that involve a risk of infection.

If the fit of a respirator is disrupted, leakage can occur via penetration of the filter, the face seal, or the exhalation valve [25]. However, only N95 filters were used in this study, and leakage via the face seal is the major source of particle penetration when using a respirator [8]. In this context, fold-type respirators have a flexible sealing surface whereas the cup-type respirators do not, so users may be able to manipulate the fold-type respirator more easily to achieve a better face seal. However, fold-type and cup-type respirators have similar face seal areas [26]. Interestingly, the nosepiece of a respirator helps to prevent leak via the face seal in the nasal area, where leaks are frequently detected [27]. In this study, the nosepiece of the fold-type respirator was freely flexible but that of the cup-type respirator was not; this difference may have contributed to the difference in the amount of leak between the two types of respirator. For example, facepiece respirators are equipped with nonadjustable head straps for face sealing, and the cup-type respirators have head straps that are longer, wider, and thicker than the fold-type and valve-type respirators [26]. Moreover, the pressure generated by the head straps could influence the fit of the respirator. For example, Niezoda et al. found that fold-type and cup-type respirators had similar fit factor values at the lower seal pressure generated by the head strap [26]. Therefore, the characteristics of the respirator, i.e., the shape of the sealing surface, type of nosepiece, type of head strap apparatus, and type of valve used to reduce exhalation resistance, could influence the final fit of the respirator.

During outbreaks of infectious disease and bioterrorism attacks, it may be necessary to wear respirators for prolonged periods of time. Rebmann et al. reported that although long-term use of a respirator did not result in a clinically relevant physiologic burden for the wearer, it was associated with many subjective symptoms [28]. Thus, compliance may be determined by selection of a respirator that is comfortable for the wearer. In this context, low-pressure facial seal areas are more comfortable to wear but are prone to leakage, whereas high-pressure facial seal areas cause discomfort that negatively affects wearer compliance [26]. Compared with fold-type respirators, cup-type respirators are more rigid and include a head strap that generates higher pressure, which could increase facial discomfort.

The protection afforded by cup types was higher when intubations were performed using the GVL and IGEL than when performed using the Macintosh laryngoscope. When our subjects were asked to choose the airway device they would prefer to use when performing an intubation associated with a risk of infection, the GVL was the preferred airway device, followed by the IGEL and Macintosh laryngoscope. Ease of use and prior clinical experience could have influenced the subjects’ preferences for particular airway devices.

This study has several limitations. First, we included only the three types of respirators used during the South Korean Middle East respiratory syndrome outbreak, i.e., cup-type and fold-type respirators with an N95 rating. Clinical trials with other respirators are needed to confirm the effect of the movements required during intubation according to the type of airway device used. Second, we only recruited emergency physicians from one emergency medical center. These individuals had variable levels of knowledge and experience of wearing respirators and using airway devices, which might have influenced our results despite inclusion of education regarding correct use of a respirator before the tests. Fourth, the intubations were performed using a Laerdal airway management trainer, which may not reflect real-life clinical situations, despite being a high-fidelity manikin.

## Conclusions

Our findings indicate that the different movements performed during endotracheal intubation using different airway devices can influence the degree of protection afforded by some respirators. Emergency physicians should consider the effects of airway device types on fit factor of N95 respirators, when they perform intubation at risk of infection.

## Supporting information

S1 File The consort checklist of information to include when reporting a randomized trial assessing non-pharmacologic treatments (NPTs). (PDF)

S2 File Hanyang university seoul hospital institutional review board: HYUH 2015-11-019-004. (DOCX)

S3 File The Clinical Research Information Service (cris.nih.go.kr: KCT0001803). (XLSX)

S4 File Minimal data set of manuscripts. (DOCX)

S5 File Database about comparing differences according to airway devices. (SAV)

S6 File Report about comparing differences according to airway devices. (DOCX)

S7 File Report about data were normal distributed or not. (DOCX)

S8 File Report about comparing differences according to N95 types. (DOCX)
