# Supplementary material for: Protection afforded by respirators when performing endotracheal intubation using a direct laryngoscope, GlideScope®, and i-gel® device: A randomized trial
Source: PLoS One. 2018 Apr 19;13(4):e0195745. doi: 10.1371/journal.pone.0195745 (PMC5909605; doi:10.1371/journal.pone.0195745)
Supplement: S2 File — (DOCX) [file pone.0195745.s002.docx]

**This protocol was written and approved local IRB (hanyang university IRB) before start clinical study.**

**Translated text was marked using blue text.**

**Clinical trials was also approved before starting clinical study.**

**IRB and clinical trials were sited on manuscripts on method part.**

**We performed study according to IRB and clinical trials protocols.**

**We translated to English main study protocol.**

**With best regards.**

연구계획서 **(study protocol)**

1. 연구제목 **(Title)**

(한글) Direct laryngoscopy, Glidescope® 및 i-gel®을 사용하여 기관 삽관 시 N95마스크의

밀착계수의 비교

(영문) Comparison of Glidescope®, Macintosh laryngoscopes and i-gel® for N95 mask performance during intubation: a randomized simulation study of intubation

2. 연구 실시 기관 명 및 주소 **(institutes and address)**

기관: 한양대학교 의과대학 응급의학교실

(Hanyang university, Dept. of Emergency medicine)

주소: 서울 성동구 왕십리로 222

3. 연구책임자 및 공동연구자 **(researcher)**

연구책임자: 임태호, 한양대학교 의과대학 응급의학교실 교수

공동연구자: 이상현, 한양대학교병원 응급의학과 임상강사

안치원, 한양대학교병원 응급의학과 전임의사

오재훈, 한양대학교 의과대학 응급의학교실 조교수

4. 연구의뢰자명 및 주소: 연구 책임자 동일

5. 연구계획 **(study protocol)**

1) 연구목적 **(purpose)**

중동호흡곤란증후군(Middle East Respiratory Syndrome; MERS)는 높은 치사율과 항바이러스제 및 백신의 부재로 감염관리 및 치료에 어려움이 있다. **(Middle East Respiratory Syndrome shows high mortality and there are difficulties in treatment due to lack of antivirus drug and vaccine)**응급의료센터의 의료진은 한 공간에서 다수의 응급환자들의 처치를 시행해서 감염의 위험에 노출되어 있다. **(Staffs engaged in emergency medical center could be exposed to infections due to many patients and isolated emergency room)** 특히, 신속한 기관 삽관이 필요한 심정지 및 호흡부전 시, 감염의 위험에 대비할 시간이 부족하고, 특히 심정지 시에는 흉부압박을 하는 동안 기관 삽관을 하여 노출의 위험이 증가할 것으로 생각된다. **(The risk of infection could be increased when staffs performed emergency endotracheal intubation (cardiac arrest, respiratory failure) due to invasive procedure (chest compression, intubation) and lack of time to manage the risk of infection)** 직접후두경은 흔하게 사용되는 기관 삽관 장치로서, 삽관 시 성대의 직접적인 노출 및 시야축의 정렬이 필요 하다.^1^ **(Direct laryngoscope is widely used endotracheal dice and it is needed to expose vocal cord, directly and arrange axis of sight.1)**의료진은 구강을 통해서 성대를 봐야 하기 때문에 호흡기를 통한 감염의 위험이 있다. **(Healthcare providers could be exposed to the risk of infection due to need to see vocal cord through oral cavity)** GlideScope® (Verathon®, Bothell, WA, USA)은 후두경 날 끝에 설치된 video camera를 이용하여, 성대를 노출시킨 후 비디오 화면을 보면서 기관 내관을 삽입하고,^2^ 성문 위 기도장비인 I-gel®은 구강을 통한 성대의 노출 없이 맹목적으로 삽입을 시행하여, 의료진의 감염노출 위험이 더 낮을 것으로 생각된다. **(GlideScope® (Verathon®, Bothell, WA, USA) has blades equipped camera on tip of blades, staffs perform intubation seeing screen 2, I-gel® is inserted blindly without exposure of vocal cord, we thought the risk of infections could be decreased if staffs use these 2 devices)** 기관 삽관 시에 널리 사용되고 있는 N95 마스크를 착용하여도, 동작에 의해서 혹은 적절하지 않은 착용에 의해서 누출의 위험이 있다.^3^  **(There could be risk of infection, even if wearing widely used N95 respirators, due to movement and improper method of wearing respirators 3)** 후두경의 종류에 따른 기관 삽관의 결과 및 호흡보호구의 성능에 대한 연구는 있었으나, 후두경의 종류에 따른 호흡보호구의 성능에 대한 연구는 없었다. **(There were studies about results of endotracheal intubations according to airway types and efficacy of respirators, there are no study bout protective performance of respirators according to types of laryngoscopes)** 기관 삽관 장비에 따라서 기관 삽관을 위한 행동 및 소요되는 시간이 달라서 밀착계수에 차이가 있을 것으로 생각하였다. **(We thought that differences of movement and time according to airway devices could make difference of fit factor)**이에 연구자는 기관 삽관 장비의 종류에 따른 N95마스크의 보호효과에 대하여 알기 위해서 이번 연구를 수행하였다. **(We performed this study to know protective performances of N95 respirators according to types of airway devices)**

2) 연구내용 (범위 및 방법) **(study method)**

2)-1.Study design

이 연구는 randomized cross-over study 이다. 2016년 01 월에 본원 IRB에서 심사를 받고, clinical trials에 등록할 예정이다. **(This study is randomized cross-over study. This study will be reviewed IRB of hanyang university at January in 2016, and we will register in clinical trials)**

2)-2. Equipment and materials

3가지 종류의 N95 filtering-facepiece respirators: 3M 1860(Cup-type); 3M 1870(Fold-type); 3M 9332(Fold with valve) 을 착용한 상태에서 직접 후두경, 비디오 후두경, 성문 외 장비와 제조사의 스타일렛과 6.5mm 내경의 기관삽관 튜브(Portex, St. Paul, MN, USA)을 사용하여 마네킨에 기관삽관을 하였다. **(Participants perform intubation using direct laryngoscope, video-laryngoscope, i-gel wearing N95 filtering-facepiece respirators: 3M 1860(Cup-type); 3M 1870(Fold-type); 3M 9332(Fold with valve)** 직접 후두경은 Macintosh laryngoscope (MCL)을 이용하였고, 비디오후두경은 angulated blade type인 GlideScope® (GVL) (Verathon®, Bothell, WA, USA)과 GlideRite® Rigid Stylet을 사용하였다. **(We used direct laryngoscope: Macintosh laryngoscope (MCL), video-layngoscope: angulated blade type인 GlideScope® (GVL) (Verathon®, Bothell, WA, USA), GlideRite® Rigid Stylet, I-gel® (IGL) (Intetsurgical Ltd., Wokingham, Berkshire, UK))** 이 실험에서 high fidelity manikin (SimMom®, Laerdal, Stavanger, Norway)에 기관 삽관을 하였다. **(We performed intubation using high fidelity manikin (SimMom®, Laerdal, Stavanger, Norway))**호흡보호구 밀착도 검사는 PortaCountR Plus (TSI,Inc., St. Paul, Minn.) with re-donning between each test를 이용하여서 마스크의 밀착도를 측정하였고, 마스크의 밀착도는 주변 환경의 미세먼지의 농도와 마스크 안의 미세먼지 농도의 비율을 이용하여 측정하였다. **(Fit test of respirators was done using PortaCountR Plus (TSI,Inc., St. Paul, Minn.) with re-donning between each test, and fit factor was calculated using ratio no. of particles in and out of respirators)**

2)-3. Participants

2016년 1월 동안 한양대학병원의 응급의료센터에서 근무하는 25명 응급의학과 의사를 모집한다. **(We will recruit 25 emergency physicians engaged in emergency medical center of hanyang university in January 2016)** 16세에서 60세 사이의 건강하고, 직접 후두경을 사용하여 기관삽관의 경험이 50회 이상인 응급의학과 의사를 실험에 참여 킨다. **(Emergency physicians (Age 16 to 60) who is healthy and has experiences of endotracheal intubation using direct laryngoscope up to 50 times participated in this study.)** 표본의 수는 G-power 3.1.2® (Heine Heinrich University, Düsseldorf, German)를 이용하였고, F tests ANOVA: Repeated measures, between factors로 분석하였다. (Sample sizes were estimated using **(G-power 3.1.2® (Heine Heinrich University, Düsseldorf, German) and F tests ANOVA: Repeated measures, between factors was used.)**후두경의 종류에 따른 N95 마스크의 밀착계수의 변화를 측정하기 위해서 시행한 이전 연구에서 밀착 계수의 평균 (표준 편차)는 MCL 128.71(41.07), GVL이 170.27 (39.25) 이고, 161.46 (38.43) 이었다. **(Pilot study to know differences of fit factor of N95 respirators according to laryngoscopes types was done and mean(SD) of fit factors were )MCL 128.71(41.07), GVL이 170.27 (39.25), igel161.46 (38.43))** α 값은 0.05, 검정력 (1-β)는 0.8로 하였다. (α values was 0.05, (1-β) was 0.8.) SD within each group (pooled SD)는 39.6이었고, Effect size f는 0.4515279이고 number of group는 3, number of measurements는 3, correlation among repeated measures는 0.15이었다. **(SD within each group (pooled SD)was 39.6, Effect size f was 0.4515279, number of group was 3, number of measurements was 3, correlation among repeated measures was 0.15.)** 계산된 Total sample size는 24명이었고, Drop rate 10%를 고려하여서 본 연구에 최소한 26명의 참가자가 필요하였다. **(Calculated Total sample size was 24, considering Drop rate 10%, 26 participants was needed.)** 모든 참여자는 자발적 의지에 따라 실험에 참여 하였고, 손목 혹은 허리의 통증을 호소하는 경우에는 실험에서 배제하였다. **(All participants participated this study according to their free will and participants who complaint wrist and lower back pain were excluded)** 실험에 참여하기 전에, 실험에 대한 설명 및 인적 사항, 기관 삽관의 경험 및 호흡보호장비의 교육 여부에 대하여 조사 한다. **(Prior to start study, survey about demography, experience of intubation and education about respirators and education about study were done)**

2)-4. Interventions

모든 참여자는 3종류의 후두경을 이용하여 기관삽관을 하고. 기관삽관의 순서는 난수 생성 사이트(www.random.org)를 이용하여서 임의할당을 한다. **(All participants performed intubation using 3 types airway devices, and order of intubation was randomly assigned using random number generating site (www.random.org))** 각각의 참가자는 3종류의 N95 마스크를 착용한 상태에서 마스크 당 3회씩 총 9회의 기관삽관을 시행한다. **(Each participant performed 3 times of intubation wearing 3 mask types, and finally each participant performed endotracheal intubation 9 times)** 기관삽관은 임상에서 기관삽관 상황을 반영하여 작성한 기관삽관 시뮬레이션 프로토콜에 따라 시행한다. **(Endotracheal intubation was performed according to simulation protocol considering clinical situations)**

각각의 참가자는 실험에 참여하기 전에 N95 mask의 착용법 및 각각의 후두경의 사용법에 대해 교육 및 실제 연습을 시행 한다. **(Each participant practice wearing N95 respirator and intubation using 3 devices and education was done prior to participate study)** 호흡보호구 밀착도 검사는 기관삽관을 시작할 때부터 기관 삽관을 시행하고 첫 번째 bag valve mask ventilation을 완료할 때까지 시행한다. **(Fit test of respirator was done from starting intubation to first bag valve mask ventilation.)** 기관 삽관 시간은 구강에 후두경을 삽입할 때부터 성대가 최대한 노출될 때까지, 노출 이후에 1번째 mask ventilation을 완료할 때까지를 측정하고, 각 시점은 참가자가 말한 것을 기록한다. **(Intubation time was measured from inserting laryngoscope blade into oral cavity to maximum vocal cord exposure and from maximum vocal cord exposure to 1^st^ mask ventilation according to participant announce)** 각각의 기관 삽관 시도 사이에는 5분간의 휴식시간이 주어진다. **(There were about 5 minute rests between each intubation trials.)**

2)-5. Outcome

본 연구의 Primary outcome은 밀착 계수 (Fit factor)이다. **(Primary outcomes is fit factor)** 밀착 계수는 (i) 구강에 후두경을 삽입 시점부터1번째bag valve mask ventilation까지 (ii)구강에 후두경을 삽입시점부터 최대한 성대를 노출할 때까지 (iii) 최대한 성대를 노출한 때부터 1번째 bag valve ventilation할 때로 나누어서 측정한다. (Fit factors were measured 3 period; **( i) From inserting laryngoscope to 1^st^ bag valve mask ventilation ii) From inserting laryngoscope to maximum vocal cord exposure, iii) From maximum vocal cord exposure to 1st bag valve mask ventilation. )** Secondary outcome으로 기관 삽관의 성공여부와 참가자의 선호도를 조사한다. **(Secondary outcomes were success rate of intubation and preferences)**

2)-6. Data analysis

자료는 정형적인 스프레드스트 어플리케이션인 (Excel, Microsoft, Redmond, WA, USA)을 사용하여 정리한다. **(Data collection was done using commercial spread sheet application (Excel, Microsoft, Redmond, WA, USA))** 자료의 분석은 the Statistical Package for the Social Sciences (SPSS) 18.0 KO for Windows (SPSS Inc., Chicago, IL, USA)를 사용하여 시행한다. **(Analysis of data was done using the Statistical Package for the Social Sciences (SPSS) 18.0 KO for Windows (SPSS Inc., Chicago, IL, USA))** 기술적 통계를 시행하고, 명목형 변수는 frequencies 와 percentages로 표시한다. **(Descriptive analysis will done, binary variables will written using frequencies and percentages.)** 연속형 변수는 정규분포를 따를 경우 Mean과standard deviation (SD)으로 기술하고, 정규분포를 따르지 않을 경우에는 a median with interquartile range (IQR)로 기술한다. **(In continuous variables, Mean and standard deviation will be used if variable follow normal distribution, and a median with interquartile range (IQR) will be used if variable did not follow normal distribution)** 정규분포를 따르는 연속형 변수는The repeated measure analysis of variance (Repeated Measures ANOVA) test 를 사용하여 분석한다. **(In continuous variables, the repeated measure analysis of variance (Repeated Measures ANOVA) will be used if variable follow normal distribution)** Mauchly's test of sphericity를 사용한다. **(Mauchly's test of sphericity)** A post-hoc analysis 는a paired t-test를 사용한다. **(A post-hoc analysis was done using a paired t-test)** 연속형 변수 중 정규분포를 따르지 않는 경우The Friedman test를 사용한다. **(In continuous variables, the Friedman test will be used if variable do not follow normal distribution)** A post-hoc 는the Wilcoxon signed-rank test로 시행하고, a Bonferroni correction을 한다. **(A post-hoc will be done using the Wilcoxon signed-rank test, a Bonferroni correction will be done.)** P value가 0.05 이한인 경우 통계적으로 유의하다고 정의한다. **(We will define that P value under 0.05 will be statistically significant)**

3) 연구의 기대효과 **(Expectation)**

이번 연구를 통해서 기관삽관 장비의 종류에 따른 의료진의 감염 위험의 노출 정도 및 마스크의 보호효과를 알고자 한다. **(We want to know the effect of airway devices types on the degrees of infection risk and protective performance of respirators)** 감염의 위험이 있는 환자의 안전한 기도 확보의 방법에 대한 중요한 의견제시 및 증거가 될 수 있다고 생각된다. **(This study could be an opinion and evidence about safely method about securing airway of patient with risk of infections)**

4) 연구추진계획 **(Plan)**

2015. 12 연구계획서 작성 및 통계전문가와 피험자의 수 산정 및 분석방법 논의 **(make a study plan and conference)**

2016. 01 기관 IRB 심의 **(IRB approve)**

2016. 01 - 2016. 02마네킨 시뮬레이션 수행 및 자료수집 **(perform simulation study and collecting data)**

2016. 02 - 2016. 4 자료 분석 **(data analysis)**

2016. 4 - 2016. 10 논문 작성 **(writing manuscript)**

2016. 10- 2016. 11 논문 완성 및 투고 (complete writing manuscript)

5) 참고 문헌 **(references)**

1. Adnet F, Borron SW, Dumas JL, Lapostolle F, Cupa M, Lapandry C. Study of the "sniffing position" by magnetic resonance imaging. Anesthesiology 2001; 94(1): 83-6.

2. Paolini JB, Donati F, Drolet P. Review article: video-laryngoscopy: another tool for difficult intubation or a new paradigm in airway management? Can J Anaesth. 2013; 60: 184-91.

3. Rengasamy S, Walbert GF, Newcomb WE, Faulkner K, Rengasamy MM, Brannen JJ, et al. Total inward leakage measurement of particulates for N95 filtering facepiece respirators--a comparison study. Ann Occup Hyg. 2014; 58(2): 206-16.

6. 연구 기간: IRB승인일로부터 ~ 2016. 11. 30 **(Schedule)**

7. 연구의 윤리적 고려사항 **(Ethical consideration)**

1) 본 연구는 헬싱키 선언, ICG-GCP, KGCP, 생명윤리 및 안전에 관한 법률 등 임상시험 관련 법규 와 기관에서 정해진 절차를 준수하면서, 피험자의 안전을 최우선으로 하여 임상시험을 윤리적이고 과학적으로 시행한다. 연구에 참여하는 피험자의 안전에 관해 면밀한 주의를 기울이며, 이상반응 등 피험자의 건강 및 안전에 문제가 발생한 경우 성실히 최선을 다해 치료 및 대처하며 피험자의 피해가 최소화 할 수 있도록 모든 노력을 다하며 피험자의 개인 정보(병원 등록번호, 주민등록번호, 성명, 주소, 전화번호 등) 및 정보 노출 시 피험자의 명성과 사생활 침해 및 직업/ 보험 등에 문제가 생길 수 있는 민감한 정보들이 연구 자료에 포함되지 않도록 하고, 부득이하게 수집된 경우 이들 정보가 외부에 노출되지 않고 보호될 수 있도록 기록지 일련번호, 피험자 식별코드 등으로만 분류하는 등 최선의 조치를 취한다.

2) 피험자를 위한 비밀보장 **(privacy policy)**

피험자의 신원을 파악할 수 있는 기록은 비밀로 보장될 것이며, 연구의 결과가 출판될 경우에도 피험자의 신원을 비밀로 유지한다. 연구에 관련된 모든 서류에는 피험자의 이름이 아닌 기록지 일련번호와 피험자 식별코드(피험자의 이니셜)만으로 기록하고 구분한다.

3) 피험자 설명서 및 동의서: 첨부 **(Manual and agreement)**

| 계획서 요약 **(Summary)** | | | | | | | | | |
| --- | --- | --- | --- | --- | --- | --- | --- | --- | --- |
| 과제명 | (한글) Direct laryngoscopy, Glidescope® 및 i-gel®을 사용하여 기관 삽관 시 N95  마스크의 밀착계수의 비교  (영문) Comparison of Glidescope®, Macintosh laryngoscopes and i-gel® for N95 mask performance during intubation: a randomized simulation study of intubation | | | | | | | | |
| 시험책임자 | 임태호, 한양대학교 의과대학 응급의학교실 교수 | | | | | | | | |
| 시험담당자 | 이상현, 한양대학교 병원 응급의학과 임상강사 | | | | | | | | |
| 실시기관명  및 주소 | 한양대학교 응급의학교실 (서울 성동구 왕십리로 222) | | | | | | | | |
| 다기관 공동연구 | □국제 □국내 | | | | | | | | |
|  | 참가국가수 개국 | | 참가기관수 개 | | | | | 국내기관 개 센터 | |
|  | □전체 P.I | 국가명: | | | | 센터명: | | | 책임자명: |
|  | □국내 P.I | 기관명: | | | | | | | 책임자명: |
| 관리약사  (의료기기관리자) |  | | | | | | | | |
| 공동연구기관 | 없음 | | | | | | | | |
| 목적 | 응급의료센터의 의료진은 감염의 위험에 노출되어 있다. 특히 중증 환자의 기관삽관시에 노출의 위험이 증가한다. 흔하게 사용되는 직접후두경은 의료진이 성대의 직접적인 노출이 필요해서 감염의 위험이 높다. GlideScope® (Verathon®, Bothell, WA, USA)은 후두경 날 끝에 설치된 video camera를 이용하여, 성대를 노출시킨 후 비디오 화면을 보면서 기관 내관을 삽입하고, 성문 위 기도장비인 I-gel®은 구강을 통한 성대의 노출 없이 맹목적으로 삽입을 시행하여, 의료진의 감염노출 위험이 더 낮을 것으로 생각된다.. 기관 삽관 장비에 따라서 기관 삽관을 위한 행동 및 소요되는 시간이 달라서 밀착계수에 차이가 있을 것으로 생각하였다. 이에 연구자는 기관 삽관 장비의 종류에 따른 N95마스크의 보호효과에 대하여 알기 위해서 이번 연구를 수행하였다. | | | | | | | | |
| 연구설계 개요 | 연구 내용  본 연구에 16세에서 60세 사이의 건강하고, 직접 후두경을 사용하여 기관삽관의 경험이 50회 이상인 응급의학과 의사가 참여한다. 기관삽관의 순서는 난수 생성 사이트(www.random.org)를 이용하여서 임의할당을 한다. 참가자는 3가지 종류의 N95 filtering-facepiece respirators: 3M 1860(Cup-type); 3M 1870(Fold-type); 3M 9332(Fold with valve) 을 착용한 상태에서 직접 후두경: Macintosh laryngoscope (MCL), 비디오 후두경: GlideScope® (GVL) (Verathon®, Bothell, WA, USA)과 제조사의 스타일렛(GlideRite® Rigid Stylet), 성문 외 장비 I-gel® (IGL) (Intetsurgical Ltd., Wokingham, Berkshire, UK)와 과 6.5mm 내경의 기관삽관 튜브(Portex, St. Paul, MN, USA)을 사용하여 마네킨에 기관삽관을 한다. 이 실험에서 기관 삽관을 구현하기 위해서 high fidelity manikin (SimMom®, Laerdal, Stavanger, Norway)을 이용한다. 호흡보호구 밀착도 검사는 PortaCountR Plus (TSI,Inc., St. Paul, Minn.) with re-donning between each test를 이용하여 시행한다.  각각의 참가자는 실험에 참여하기 전에 N95 mask의 착용법 및 각각의 후두경의 사용법에 대해 교육 및 실제 연습을 시행 하였다. 호흡보호구 밀착도 검사는 기관삽관을 시작할 때부터 기관 삽관을 시행하고 첫 번째 bag valve mask ventilation을 완료할 때까지 시행하였다. 기관 삽관 시간은 구강에 후두경을 삽입할 때부터 성대가 최대한 노출될 때까지, 노출 이후에 1번째 mask ventilation을 완료할 때까지를 측정하였고, 각 시점은 참가자가 말한 것을 기록하였다. 각각의 기관 삽관 시도 사이에는 5분간의 휴식시간이 주어진다.  Outcome  본 연구의 Primary outcome은 밀착 계수 (Fit factor)이다. 밀착 계수는 (i) 구강에 후두경을 삽입 시점부터1번째bag valve mask ventilation까지 (ii)구강에 후두경을 삽입시점부터 최대한 성대를 노출할 때까지 (iii) 최대한 성대를 노출한 때부터 1번째 bag valve ventilation할 때로 나누어서 측정하였다. Secondary outcome으로 기관 삽관의 성공여부와 참가자의 선호도를 조사한다. | | | | | | | | |
| 시험약(임상시험용의료기기) | 사용없음 | | | | | | | | |
| 대조약*  (임상시험용의료기기) | 사용없음 | | | | | | | | |
| 대상질환명 | 없음 | | | 상병명 | | | 없음 | | |
| 연구의 특성 | 피험자 모집 방법 , 자발적 참여 O  피험자 모집 문건(광고, 전단, 인터넷, 이메일 등)의 사용 □ 예 ■ 아니오  (사용 전 반드시 IRB 심의를 받고 승인을 득하여야 함)  피험자와 접촉 여부 ■ 예 □ 아니오  시술 / 투약 / 검사 등의 중재 여부 □ 예 ■ 아니오  피험자의 사적 정보(의무 정보 등)의 이용 여부 ■ 예 □ 아니오  시료의 수집 및 보관 여부 □ 예 ■ 아니오  유전학적 정보의 수집 및 보관 여부 □ 예 ■ 아니오  사용되는 시술 □ 침습적 □ 비침습적 ■ 해당 사항 없음  다기관 공동연구 □ 예 ■ 아니오  경제적 이해관계 명시 ■ 해당사항 없음 □ 해당사항 있음________________ | | | | | | | | |
| 피험자수 | 본 기관 배정 25 명 | | | | 전체 25명(국내: 25 명, 국외 0 명) | | | | |
|  | 산출 근거 * 표본의 수는 G-power 3.1.2® (Heine Heinrich University, Düsseldorf, German)를 이용하였고, F tests ANOVA: Repeated measures, between factors로 분석하였다. 후두경의 종류에 따른 N95 마스크의 밀착계수의 변화를 측정하기 위해서 시행한 이전 연구에서 밀착 계수의 평균 (표준 편차)는 MCL 128.71(41.07), GVL이 170.27 (39.25) 이고, 161.46 (38.43) 이었다. α 값은 0.05, 검정력 (1-β)는 0.8로 하였다. SD within each group (pooled SD)는 39.6이었고, Effect size f는 0.4515279이고 number of group는 3, number of measurements는 3, correlation among repeated measures는 0.15이었다. 계산된 Total sample size는 24명이었고, Drop rate 10%를 고려하여서 본 연구에 최소한 26명의 참가자가 필요하였다. | | | | | | | | |
| 피험자  정보 | 연구 대상군: ■ 건강인 □ 환자 □ 취약한 피험자 군 | | | | | | | | |
|  | 취약한 피험자 범주(□에 ∨)  □임산부 영/소아  □장애인 (□ 육체적 □ 인지적 □ 정신적)  □연구기관, 책임 연구자,의뢰자 등의 피고용인 ■ 책임연구자의 연구원이나 학생  □학교의 학생 □ 군인 또는 군대 조직에 의한 피험자 모집  □시설에 수용된 자 □ 수감자 □ 외국인  □말기 환자 □ 사회적 낙인이 찍힌 질환을 가진 자  윤리적 대책: 피실험자는 실험에 참여하기 전에 실험의 절차 및 참여로 인해서 발생할 수 있는 불편함에 대하여 설명을 듣고 동의서를 작성합니다. 피험자가 원할 시 언제든 동의 철회가 가능하고, 참여로 인한 불편함이 발생 시 즉시 실험을 중단할 수 있음을 사전에 설명할 것입니다. | | | | | | | | |
| 선정기준 | 2015년10월 동안 응급의료센터에서 18세에서 60세 사이의 건강하고, 직접 후두경을 사용한 기관삽관의 경험이 50회 이상인 응급의학과 의사를 모집한다. | | | | | | | | |
| 제외기준 | 손목 혹은 허리의 통증을 호소하는 경우에는 실험에서 배제한다. | | | | | | | | |
| 스크리닝 |  | | | | | | | | |
| 연구방법 | . 3가지 종류의 N95마스크를 착용하고, 3가지 종류의 후두경으로 기관삽관을 하면서 실시간으로 밀착 계수를 측정하다. | | | | | | | | |
| 유효성 평가  항목 및 방법 |  | | | | | | | | |
| 통계분석방법 | 자료는 정형적인 스프레드스트 어플리케이션인 (Excel, Microsoft, Redmond, WA, USA)을 사용하여 정리한다. 자료의 분석은 the Statistical Package for the Social Sciences (SPSS) 18.0 KO for Windows (SPSS Inc., Chicago, IL, USA)를 사용하여 시행하였다. 기술적 통계를 시행하고, 명목형 변수는 frequencies 와 percentages로 표시하였다. 연속형 변수는 정규분포를 따를 경우 Mean과standard deviation (SD)으로 기술하고, 정규분포를 따르지 않을 경우에는 a median with interquartile range (IQR)로 기술한다. 정규분포를 따르는 연속형 변수는The repeated measure analysis of variance (Repeated Measures ANOVA) test 를 사용하여 분석한다. Mauchly's test of sphericity를 사용한다. A post-hoc analysis 는a paired t-test를 사용한다. 연속형 변수 중 정규분포를 따르지 않는 경우The Friedman test를 사용한다. A post-hoc 는the Wilcoxon signed-rank test로 시행하였고, a Bonferroni correction을 한다. P value가 0.05 이한인 경우 통계적으로 유의하다고 정의한다. | | | | | | | | |

**설 명 서 (Manual)**

| **연 구 제 목** | **(한글)** Direct laryngoscopy, Glidescope® 및 i-gel®을 사용하여 기관삽관 시 N95마스크의  밀착계수의 비교  **(영문)** Comparison of Glidescope®, Macintosh laryngoscopes and i-gel® for N95 mask performance during intubation: a randomized simulation study of intubation |
| --- | --- |

**1. 연구목적**

응급의료센터에서 근무하는 의료진은 감염에 노출될 위험이 높습니다. 특히, 호흡부전, 의식변화 등의 응급 기관 삽관이 필요한 상황에서는 감염의 위험이 더욱 증가하여 적절한 호흡보호구의 사용이 중요합니다. 흔하게 사용되는 N95마스크는 움직임에 따라서 보호성능이 영향을 받습니다. 상용화된 기도장비로 직접후두경, 비디오 후두경, 성문 위 장비 등이 있습니다. 직접후두경이 흔하게 사용되고 있으나, 직접적인 성문의 노출이 필요하여 감염노출의 위험이 높습니다. 이에 연구진은 기도장비의 종류에 따른 N95마스크의 보호성능의 차이를 알아보고자 연구를 계획하였습니다.

**2. 연구참여 및 수행방법**

본 연구는 기관삽관의 경험이 50회 이상인 건강한 응급의학과의사를 대상으로 합니다. 참여자분 들은 실험에 참여하기 전에 실험에 대한 설명서 및 동의서를 작성합니다. 기도장비 및 N95마스크에 대한 교육을 받으신 후에 실험에 참여합니다. 3가지 종류의 N95 마스크를 착용한 상태에서, 직접 후두경, 비디오 후두경; GlideScope® (Verathon®, Bothell, WA, USA), 성문 위 장치; I-gel® 을 이용하여서 기관 삽관 용 마네킨에 3종류의 마스크를 착용하고 각각의 마스크당 3회 총 9 회의 기관삽관을 시행합니다. 각각의 기관삽관 사이에는 5분간의 휴식시간이 있습니다.

**3. 연구대상자의 연구참여 기간 및 소요되는 시간**

1일에 1회씩 총 1회 방문하며 1회 방문당 서류의 작성을 포함하여서 1시간 가량 소요 예정입니다.

**4. 자발적 참여, 자유로운 동의 철회**

연구대상자는 자발적으로 연구에 참여함을 동의하시고, 언제든지 연구대상자 분이 원하실 때 동의 철회가 가능하며, 철회 시 어떠한 불이익도 없습니다.

**5. 개인정보 수집 및 활용에 관한 사항**

만약 귀하가 본 연구에 참여하신다면 의무기록 및 귀하의 신원을 직접적으로 파악할 수 있는 정보는 신뢰할 수 있는 방법으로 보호하여 사용할 것입니다. 본 연구로부터 얻어진 자료와 정보는 출판되거나 혹은 관계 당국에 의해 직접 열람될 수 있으나 이러한 자료 혹은 정보 어디에도 귀하의 이름을 언급하지 않을 것입니다.

**6. 연구 관련 자료 보관 및 폐기에 관한 사항**

기본문서와 이 기준 및 관계 법령에 따라 작성된 전자문서를 포함한 임상시험 관련 문서를 의약품 임상시험 관리기준 제9호 및 관계 법령에 따라 보관하며 결과보고서 작성을 마친 후에는 이들 문서를 의약품 임상시험 관리기준 제5호나목8)에 따른 보관책임자에게 인계한다. 그리고, 이번 연구와 관련된 자료는 실험 종료 후 2년간 보관할 예정입니다.

**7. 이익/위험도**

이 시험의 참여로 귀하에게 직접적 혜택은 없습니다. 다만, 이 연구의 결과는 감염의 위험이 있는 환자의 안전한 기도 확보의 방법에 대한 중요한 의견제시 및 증거가 될 수 있다고 생각됩니다. 실험에 의한 직접적인 불편 혹은 위해는 없습니다. N95 마스크 착용에 따른 약간의 불편감이 있으며, 실험 참여자 분이 원하는 경우에 즉시 실험을 멈출 것입니다.

**8. 연구참여로 인한 손실에 대한 보상**

연구참여로 인해 연구대상자 분이 할애한 시간, 이동에 따른 교통비 등에 대한 금전적인 보상은 없습니다. 연구참여자 분의 실험참여는 의료진 및 환자의 감염 노출의 예방 및 안전한 기관삽관을 하는 데 기여할 것으로 생각됩니다.

**9. 문의처**

이 연구는 본 기관 연구대상자의 권리, 안전, 복지를 보호할 책임이 있는 한양대학병원 기관생명윤리위원회(IRB)에 의해 승인되었으며, 본 연구의 참가자로서 귀하의 권익 보호 및 연구 윤리에 대한 문의가 있을 경우 한양대학병원 기관생명윤리위원회 02-2290-9653)에게 연락하실 수 있습니다. 연구에 대한 문의사항이 있거나 위험이나 불편함이 발생할 경우, 아래 연구책임자에게 연락하여 주시기 바랍니다.

| 연구책임자 | 성명 | 연락처 | 연구실시기관명 |
| --- | --- | --- | --- |
|  | 임태호 | +82 2 22909291 | 한양대학교 의과대학 응급의학과 교실 |

**동 의 서
(Agreement)**

| **연 구 제 목** | **(한글)** Direct laryngoscopy, Glidescope® 및 i-gel®을 사용하여 기관삽관 시 N95마스크의  밀착계수의 비교  **(영문)** Comparison of Glidescope®, Macintosh laryngoscopes and i-gel® for N95 mask performance during intubation: a randomized simulation study of intubation |
| --- | --- |

**◇ 본인은 설명서를 읽었으며 본 연구의 목적, 방법 등에 대한 충분한 설명을 듣고 이해하였습니다.**

**◇ 모든 궁금한 사항에 대해 질문하였고, 충분한 답변을 들었습니다.**

**◇ 본인은 설명서 및 작성된 동의서 사본 1부를 받았음을 확인합니다.**

**◇ 충분한 시간을 갖고 생각한 결과, 본인은 이 연구에 참여하기를 자유로운 의사에 따라 동의합니다.**

| **연구대상자의** | **성명** |  | **(인)** | **2015년 월 일** |
| --- | --- | --- | --- | --- |
| **법정대리인의(해당시)** | **성명** |  | **(인)** | **2015년 월 일** |
| **연구책임자의** | **성명** |  | **(인)** | **2015년 월 일** |
