# Supplementary material for: Protection afforded by respirators when performing endotracheal intubation using a direct laryngoscope, GlideScope®, and i-gel® device: A randomized trial
Source: PLoS One. 2018 Apr 19;13(4):e0195745. doi: 10.1371/journal.pone.0195745 (PMC5909605; doi:10.1371/journal.pone.0195745)
Supplement: S4 File — (DOCX) [file pone.0195745.s004.docx]

**[Minimal Data set of PONE-D-18-04954R1]**

**Protection afforded by respirators when performing endotracheal intubation using a direct laryngoscope, GlideScope®, and i-gel® device: a randomized trial**

1. **Data about sample size**

**Manuscripts**


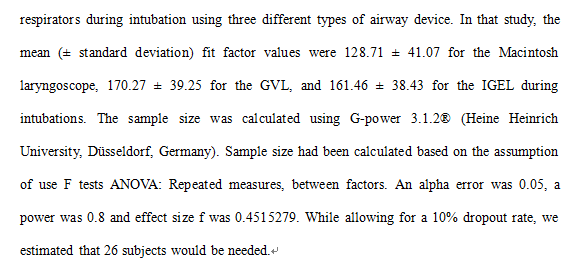


**Capture image of the result of G-power**


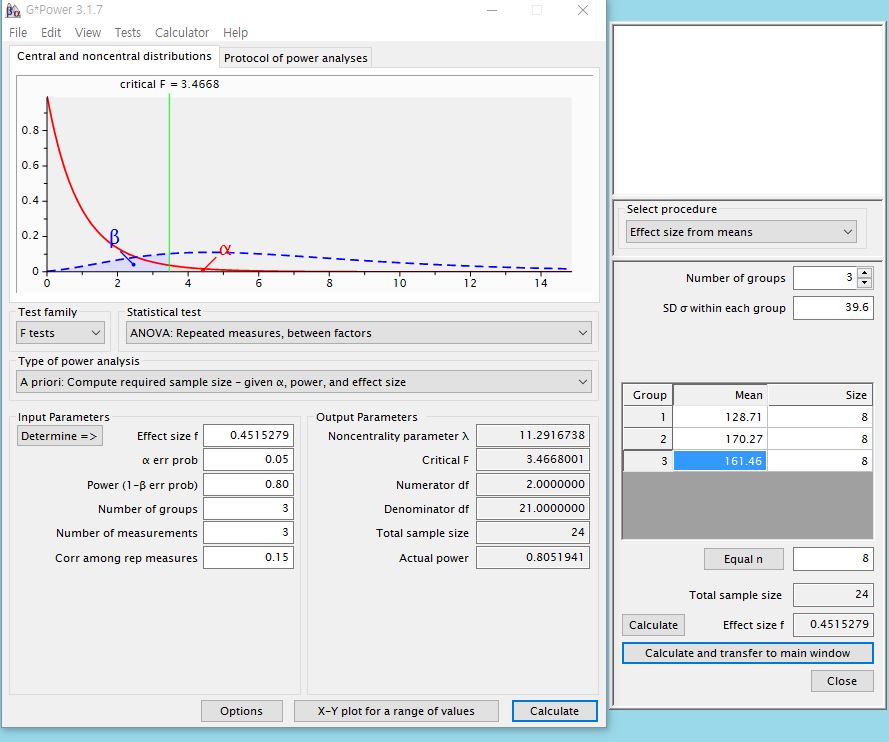


1. **Data about distribution**

- **This text is the result of SPSS. We copy the result of SPSS for easiness of reader who did not have SPSS programs.**

1. **larygo_type = 1.00, 1.cup,2fold,3valve = cup type**

**(Direct laryngoscope, Cup type)**

| **케이스 처리 요약^a^** | | | | | | |
| --- | --- | --- | --- | --- | --- | --- |
|  | 케이스 | | | | | |
|  | 유효 | | 결측 | | 전체 | |
|  | N | 퍼센트 | N | 퍼센트 | N | 퍼센트 |
| mask_fittest | 441 | 100.0% | 0 | 0.0% | 441 | 100.0% |
| a. larygo_type = 1.00, 1.cup,2fold,3valve = cup type | | | | | | |

| **기술통계^a^** | | | | |
| --- | --- | --- | --- | --- |
|  | | | 통계량 | 표준오차 |
| mask_fittest | 평균 (Mean) | | 136.8451 | 3.24751 |
|  | 평균의 95% 신뢰구간 | 하한 | 130.4626 |  |
|  |  | 상한 | 143.2277 |  |
|  | 5% 절삭평균 | | 140.0322 |  |
|  | 중위수 (Median) | | 166.0000 |  |
|  | 분산 | | 4650.919 |  |
|  | 표준편차 | | 68.19764 |  |
|  | 최소값 | | .00 |  |
|  | 최대값 | | 200.00 |  |
|  | 범위 | | 200.00 |  |
|  | 사분위수 범위 | | 130.00 |  |
|  | 왜도 | | -.479 | .116 |
|  | 첨도 | | -1.397 | .232 |
| a. larygo_type = 1.00, 1.cup,2fold,3valve = cup type | | | | |

| **정규성 검정^a^** | | | | | | |
| --- | --- | --- | --- | --- | --- | --- |
|  | Kolmogorov-Smirnov^b^ | | | Shapiro-Wilk | | |
|  | 통계량 | 자유도 | 유의확률 | 통계량 | 자유도 | 유의확률 |
| mask_fittest | .263 | 441 | .000 | .808 | 441 | .000 |
| a. larygo_type = 1.00, 1.cup,2fold,3valve = cup type | | | | | | |
| b. Lilliefors 유의확률 수정 | | | | | | |

Not normally distributed. Because *P* vale is under 0.05

1. **larygo_type = 1.00, 1.cup,2fold,3valve = fold type**

**(Direct laryngoscope, Fold type)**

| **케이스 처리 요약^a^** | | | | | | |
| --- | --- | --- | --- | --- | --- | --- |
|  | 케이스 | | | | | |
|  | 유효 | | 결측 | | 전체 | |
|  | N | 퍼센트 | N | 퍼센트 | N | 퍼센트 |
| mask_fittest | 396 | 100.0% | 0 | 0.0% | 396 | 100.0% |
| a. larygo_type = 1.00, 1.cup,2fold,3valve = fold type | | | | | | |

| **기술통계^a^** | | | | |
| --- | --- | --- | --- | --- |
|  | | | 통계량 | 표준오차 |
| mask_fittest | 평균 | | 189.2614 | 5.02295 |
|  | 평균의 95% 신뢰구간 | 하한 | 179.3863 |  |
|  |  | 상한 | 199.1364 |  |
|  | 5% 절삭평균 | | 191.7890 |  |
|  | 중위수 | | 200.0000 |  |
|  | 분산 | | 9991.109 |  |
|  | 표준편차 | | 99.95554 |  |
|  | 최소값 | | 9.60 |  |
|  | 최대값 | | 2000.00 |  |
|  | 범위 | | 1990.40 |  |
|  | 사분위수 범위 | | .00 |  |
|  | 왜도 | | 14.913 | .123 |
|  | 첨도 | | 274.112 | .245 |
| a. larygo_type = 1.00, 1.cup,2fold,3valve = fold type | | | | |

| **정규성 검정^a^** | | | | | | |
| --- | --- | --- | --- | --- | --- | --- |
|  | Kolmogorov-Smirnov^b^ | | | Shapiro-Wilk | | |
|  | 통계량 | 자유도 | 유의확률 | 통계량 | 자유도 | 유의확률 |
| mask_fittest | .455 | 396 | .000 | .170 | 396 | .000 |
| a. larygo_type = 1.00, 1.cup,2fold,3valve = fold type | | | | | | |
| b. Lilliefors 유의확률 수정 | | | | | | |

Not normally distributed. Because *P* vale is under 0.05

1. **larygo_type = 2.00, 1.cup,2fold,3valve = cup type**

**(Glidesope, Cup type)**

| **케이스 처리 요약^a^** | | | | | | |
| --- | --- | --- | --- | --- | --- | --- |
|  | 케이스 | | | | | |
|  | 유효 | | 결측 | | 전체 | |
|  | N | 퍼센트 | N | 퍼센트 | N | 퍼센트 |
| mask_fittest | 383 | 100.0% | 0 | 0.0% | 383 | 100.0% |
| a. larygo_type = 2.00, 1.cup,2fold,3valve = cup type | | | | | | |

| **기술통계^a^** | | | | |
| --- | --- | --- | --- | --- |
|  | | | 통계량 | 표준오차 |
| mask_fittest | 평균 | | 175.7102 | 5.42265 |
|  | 평균의 95% 신뢰구간 | 하한 | 165.0482 |  |
|  |  | 상한 | 186.3722 |  |
|  | 5% 절삭평균 | | 176.8697 |  |
|  | 중위수 (Median) | | 200.0000 |  |
|  | 분산 | | 11262.159 |  |
|  | 표준편차 | | 106.12332 |  |
|  | 최소값 | | 10.00 |  |
|  | 최대값 | | 2002.00 |  |
|  | 범위 | | 1992.00 |  |
|  | 사분위수 범위 | | 48.00 |  |
|  | 왜도 | | 13.218 | .125 |
|  | 첨도 | | 230.449 | .249 |
| a. larygo_type = 2.00, 1.cup,2fold,3valve = cup type | | | | |

| **정규성 검정^a^** | | | | | | |
| --- | --- | --- | --- | --- | --- | --- |
|  | Kolmogorov-Smirnov^b^ | | | Shapiro-Wilk | | |
|  | 통계량 | 자유도 | 유의확률 | 통계량 | 자유도 | 유의확률 |
| mask_fittest | .407 | 383 | .000 | .268 | 383 | .000 |
| a. larygo_type = 2.00, 1.cup,2fold,3valve = cup type | | | | | | |
| b. Lilliefors 유의확률 수정 | | | | | | |

Not normally distributed. Because *P* vale is under 0.05

1. **larygo_type = 2.00, 1.cup,2fold,3valve = fold type**

**(Glidesope, Fold type)**

| **케이스 처리 요약^a^** | | | | | | |
| --- | --- | --- | --- | --- | --- | --- |
|  | 케이스 | | | | | |
|  | 유효 | | 결측 | | 전체 | |
|  | N | 퍼센트 | N | 퍼센트 | N | 퍼센트 |
| mask_fittest | 428 | 100.0% | 0 | 0.0% | 428 | 100.0% |
| a. larygo_type = 2.00, 1.cup,2fold,3valve = fold type | | | | | | |

| **기술통계^a^** | | | | |
| --- | --- | --- | --- | --- |
|  | | | 통계량 | 표준오차 |
| mask_fittest | 평균 | | 193.7453 | 1.26677 |
|  | 평균의 95% 신뢰구간 | 하한 | 191.2554 |  |
|  |  | 상한 | 196.2352 |  |
|  | 5% 절삭평균 | | 199.0099 |  |
|  | 중위수 (Median) | | 200.0000 |  |
|  | 분산 | | 686.813 |  |
|  | 표준편차 | | 26.20712 |  |
|  | 최소값 | | 20.00 |  |
|  | 최대값 | | 200.00 |  |
|  | 범위 | | 180.00 |  |
|  | 사분위수 범위 | | .00 |  |
|  | 왜도 | | -5.026 | .118 |
|  | 첨도 | | 26.154 | .235 |
| a. larygo_type = 2.00, 1.cup,2fold,3valve = fold type | | | | |

| **정규성 검정^a^** | | | | | | |
| --- | --- | --- | --- | --- | --- | --- |
|  | Kolmogorov-Smirnov^b^ | | | Shapiro-Wilk | | |
|  | 통계량 | 자유도 | 유의확률 | 통계량 | 자유도 | 유의확률 |
| mask_fittest | .508 | 428 | .000 | .256 | 428 | .000 |
| a. larygo_type = 2.00, 1.cup,2fold,3valve = fold type | | | | | | |
| b. Lilliefors 유의확률 수정 | | | | | | |

Not normally distributed. Because *P* vale is under 0.05

1. **larygo_type = 3.00, 1.cup,2fold,3valve = cup type**

**(i-gel, Cup type)**

| **케이스 처리 요약^a^** | | | | | | |
| --- | --- | --- | --- | --- | --- | --- |
|  | 케이스 | | | | | |
|  | 유효 | | 결측 | | 전체 | |
|  | N | 퍼센트 | N | 퍼센트 | N | 퍼센트 |
| mask_fittest | 214 | 100.0% | 0 | 0.0% | 214 | 100.0% |
| a. larygo_type = 3.00, 1.cup,2fold,3valve = cup type | | | | | | |

| **기술통계^a^** | | | | |
| --- | --- | --- | --- | --- |
|  | | | 통계량 | 표준오차 |
| mask_fittest | 평균 | | 161.1818 | 3.81464 |
|  | 평균의 95% 신뢰구간 | 하한 | 153.6625 |  |
|  |  | 상한 | 168.7011 |  |
|  | 5% 절삭평균 | | 166.1563 |  |
|  | 중위수 (Median) | | 200.0000 |  |
|  | 분산 | | 3114.014 |  |
|  | 표준편차 | | 55.80335 |  |
|  | 최소값 | | 6.80 |  |
|  | 최대값 | | 200.00 |  |
|  | 범위 | | 193.20 |  |
|  | 사분위수 범위 | | 78.75 |  |
|  | 왜도 | | -1.135 | .166 |
|  | 첨도 | | -.139 | .331 |
| a. larygo_type = 3.00, 1.cup,2fold,3valve = cup type | | | | |

| **정규성 검정^a^** | | | | | | |
| --- | --- | --- | --- | --- | --- | --- |
|  | Kolmogorov-Smirnov^b^ | | | Shapiro-Wilk | | |
|  | 통계량 | 자유도 | 유의확률 | 통계량 | 자유도 | 유의확률 |
| mask_fittest | .331 | 214 | .000 | .721 | 214 | .000 |
| a. larygo_type = 3.00, 1.cup,2fold,3valve = cup type | | | | | | |
| b. Lilliefors 유의확률 수정 | | | | | | |

Not normally distributed. Because *P* vale is under 0.05

1. **larygo_type = 3.00, 1.cup,2fold,3valve = fold type**

**(i-gel, Fold type)**

| **케이스 처리 요약^a^** | | | | | | |
| --- | --- | --- | --- | --- | --- | --- |
|  | 케이스 | | | | | |
|  | 유효 | | 결측 | | 전체 | |
|  | N | 퍼센트 | N | 퍼센트 | N | 퍼센트 |
| mask_fittest | 209 | 100.0% | 0 | 0.0% | 209 | 100.0% |
| a. larygo_type = 3.00, 1.cup,2fold,3valve = fold type | | | | | | |

| **기술통계^a^** | | | | |
| --- | --- | --- | --- | --- |
|  | | | 통계량 | 표준오차 |
| mask_fittest | 평균 | | 198.3923 | .61935 |
|  | 평균의 95% 신뢰구간 | 하한 | 197.1713 |  |
|  |  | 상한 | 199.6134 |  |
|  | 5% 절삭평균 | | 200.0000 |  |
|  | 중위수 ( Median) | | 200.0000 |  |
|  | 분산 | | 80.172 |  |
|  | 표준편차 | | 8.95390 |  |
|  | 최소값 | | 123.00 |  |
|  | 최대값 | | 200.00 |  |
|  | 범위 | | 77.00 |  |
|  | 사분위수 범위 | | .00 |  |
|  | 왜도 | | -6.250 | .168 |
|  | 첨도 | | 40.665 | .335 |
| a. larygo_type = 3.00, 1.cup,2fold,3valve = fold type | | | | |

| **정규성 검정^a^** | | | | | | |
| --- | --- | --- | --- | --- | --- | --- |
|  | Kolmogorov-Smirnov^b^ | | | Shapiro-Wilk | | |
|  | 통계량 | 자유도 | 유의확률 | 통계량 | 자유도 | 유의확률 |
| mask_fittest | .523 | 209 | .000 | .176 | 209 | .000 |
| a. larygo_type = 3.00, 1.cup,2fold,3valve = fold type | | | | | | |
| b. Lilliefors 유의확률 수정 | | | | | | |

Not normally distributed. Because *P* vale is under 0.05

**3.Statistical analysis about Fit factor**


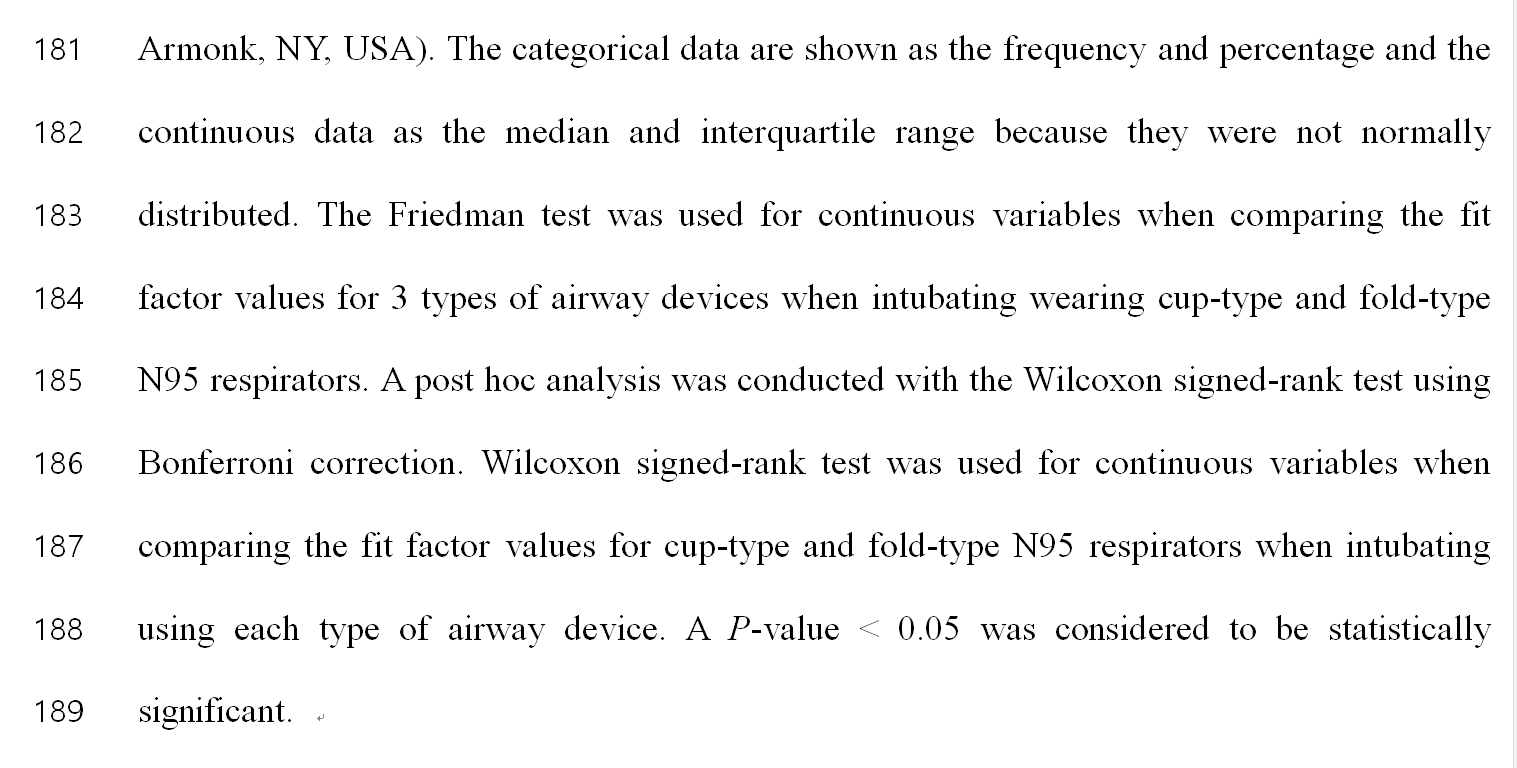


- 1. **The Friedman Test (Table 2)**
- **This text is the result of SPSS. We copy the result of SPSS for easiness of reader who did not have SPSS programs.**

**Friedman 검정**

| **순위** | |
| --- | --- |
|  | 평균순위 |
| dl_cup | 2.12 |
| dl_fold | 4.27 |
| gvl_cup | 3.09 |
| gvl_fold | 4.27 |
| igel_cup | 2.99 |
| igel_fold | 4.27 |

| **검정 통계량^a^** | |
| --- | --- |
| N | 209 |
| 카이제곱 | 448.286 |
| 자유도 | 5 |
| 근사 유의확률 | .000 |
| a. Friedman 검정 | |

- 1. **The Wilcoxon signed rank test (Table 2)**
- **This text is the result of SPSS. We copy the result of SPSS for easiness of reader who did not have SPSS programs.**

**Wilcoxon 부호순위 검정**

| **순위** | | | | |
| --- | --- | --- | --- | --- |
|  | | N | 평균순위 | 순위합 |
| gvl_cup - dl_cup | 음의 순위 | 74^a^ | 105.55 | 7810.50 |
|  | 양의 순위 | 190^b^ | 143.00 | 27169.50 |
|  | 동률 | 119^c^ |  |  |
|  | 합계 | 383 |  |  |
| igel_cup - dl_cup | 음의 순위 | 45^d^ | 76.12 | 3425.50 |
|  | 양의 순위 | 120^e^ | 85.58 | 10269.50 |
|  | 동률 | 49^f^ |  |  |
|  | 합계 | 214 |  |  |
| igel_cup - gvl_cup | 음의 순위 | 78^g^ | 65.07 | 5075.50 |
|  | 양의 순위 | 59^h^ | 74.19 | 4377.50 |
|  | 동률 | 77^i^ |  |  |
|  | 합계 | 214 |  |  |
| gvl_fold - dl_fold | 음의 순위 | 0^j^ | .00 | .00 |
|  | 양의 순위 | 0^k^ | .00 | .00 |
|  | 동률 | 396^l^ |  |  |
|  | 합계 | 396 |  |  |
| igel_fold - dl_fold | 음의 순위 | 0^m^ | .00 | .00 |
|  | 양의 순위 | 0^n^ | .00 | .00 |
|  | 동률 | 209^o^ |  |  |
|  | 합계 | 209 |  |  |
| igel_fold - gvl_fold | 음의 순위 | 0^p^ | .00 | .00 |
|  | 양의 순위 | 0^q^ | .00 | .00 |
|  | 동률 | 209^r^ |  |  |
|  | 합계 | 209 |  |  |
| a. gvl_cup < dl_cup | | | | |
| b. gvl_cup > dl_cup | | | | |
| c. gvl_cup = dl_cup | | | | |
| d. igel_cup < dl_cup | | | | |
| e. igel_cup > dl_cup | | | | |
| f. igel_cup = dl_cup | | | | |
| g. igel_cup < gvl_cup | | | | |
| h. igel_cup > gvl_cup | | | | |
| i. igel_cup = gvl_cup | | | | |
| j. gvl_fold < dl_fold | | | | |
| k. gvl_fold > dl_fold | | | | |
| l. gvl_fold = dl_fold | | | | |
| m. igel_fold < dl_fold | | | | |
| n. igel_fold > dl_fold | | | | |
| o. igel_fold = dl_fold | | | | |
| p. igel_fold < gvl_fold | | | | |
| q. igel_fold > gvl_fold | | | | |
| r. igel_fold = gvl_fold | | | | |

| **검정 통계량^a^** | | | | | | |
| --- | --- | --- | --- | --- | --- | --- |
|  | gvl_cup - dl_cup | igel_cup - dl_cup | igel_cup - gvl_cup | gvl_fold - dl_fold | igel_fold - dl_fold | igel_fold - gvl_fold |
| Z | -7.795^b^ | -5.568^b^ | -.750^c^ | .000^d^ | .000^d^ | .000^d^ |
| 근사 유의확률(양측) | .000 | .000 | .453 | 1.000 | 1.000 | 1.000 |
| a. Wilcoxon 부호순위 검정 | | | | | | |
| b. 음의 순위를 기준으로. | | | | | | |
| c. 양의 순위를 기준으로. | | | | | | |
| d. 음의 순위의 합계는 양의 순위의 합계와 같습니다. | | | | | | |

- 1. **The Wilcoxon signed rank test (Figure 3.)**
- **This text is the result of SPSS. We copy the result of SPSS for easiness of reader who did not have SPSS programs.**

**비모수 검정**


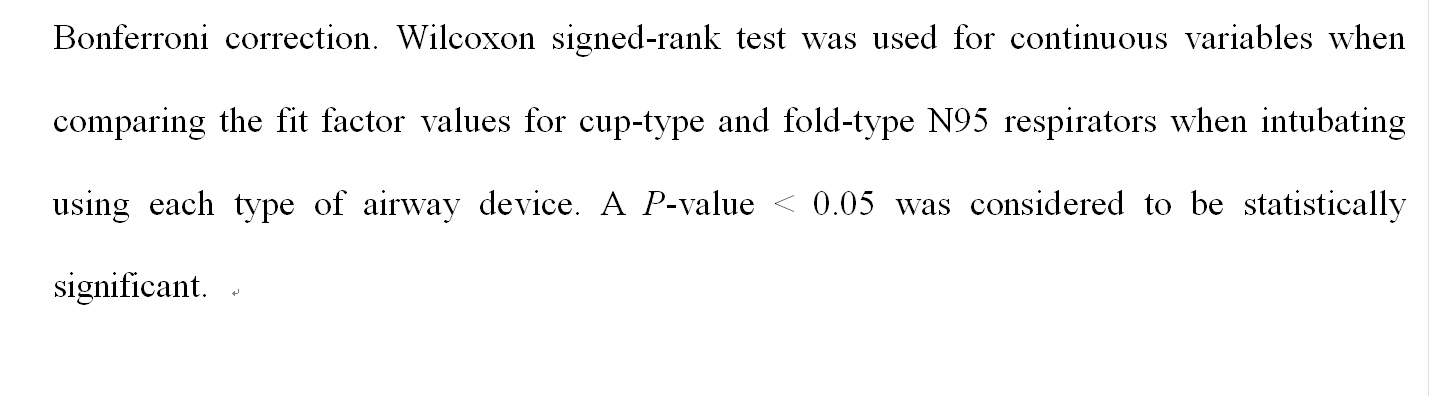


**비모수 검정**

[데이터집합0] C:\Users\user\Desktop\mask_ laryngo_friedman.sav

**Wilcoxon 부호순위 검정**

| **순위** | | | | |
| --- | --- | --- | --- | --- |
|  | | N | 평균순위 | 순위합 |
| dl_fold - dl_cup | 음의 순위 | 0^a^ | .00 | .00 |
|  | 양의 순위 | 226^b^ | 113.50 | 25651.00 |
|  | 동률 | 170^c^ |  |  |
|  | 합계 | 396 |  |  |
| gvl_fold - gvl_cup | 음의 순위 | 1^d^ | 128.00 | 128.00 |
|  | 양의 순위 | 127^e^ | 64.00 | 8128.00 |
|  | 동률 | 255^f^ |  |  |
|  | 합계 | 383 |  |  |
| igel_fold - igel_cup | 음의 순위 | 0^g^ | .00 | .00 |
|  | 양의 순위 | 90^h^ | 45.50 | 4095.00 |
|  | 동률 | 119^i^ |  |  |
|  | 합계 | 209 |  |  |
| a. dl_fold < dl_cup | | | | |
| b. dl_fold > dl_cup | | | | |
| c. dl_fold = dl_cup | | | | |
| d. gvl_fold < gvl_cup | | | | |
| e. gvl_fold > gvl_cup | | | | |
| f. gvl_fold = gvl_cup | | | | |
| g. igel_fold < igel_cup | | | | |
| h. igel_fold > igel_cup | | | | |
| i. igel_fold = igel_cup | | | | |

| **검정 통계량^a^** | | | |
| --- | --- | --- | --- |
|  | dl_fold - dl_cup | gvl_fold - gvl_cup | igel_fold - igel_cup |
| Z | -13.034^b^ | -9.513^b^ | -8.239^b^ |
| 근사 유의확률(양측) | .000 | .000 | .000 |
| a. Wilcoxon 부호순위 검정 | | | |
| b. 음의 순위를 기준으로. | | | |

**5. Median and IQR (Table 2)**

**- This text is the result of SPSS. We copy the result of SPSS for easiness of reader who did not have SPSS programs.**


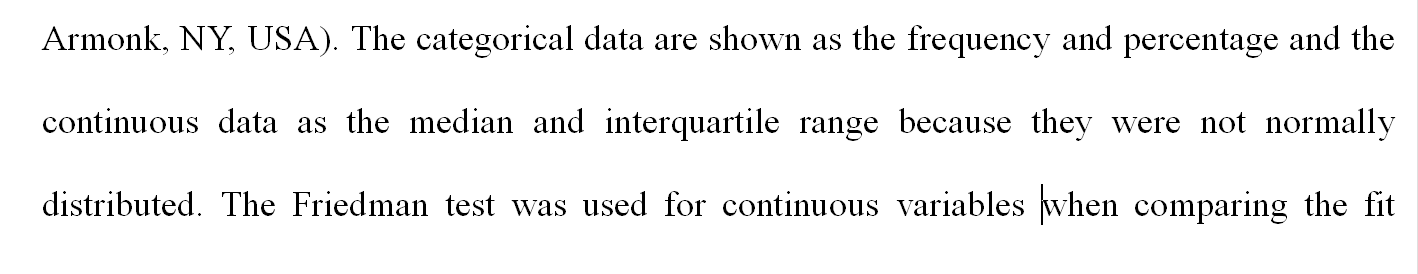


| **통계량 (Fit factor)** | | | | | | | | | |
| --- | --- | --- | --- | --- | --- | --- | --- | --- | --- |
|  | | dl_cup | dl_fold | gvl_cup | gvl_fold | igel_cup | igel_fold | base_cup | base_fold |
| N | 유효 | 441 | 396 | 383 | 428 | 214 | 209 | 292 | 234 |
|  | 결측 | 0 | 45 | 58 | 13 | 227 | 232 | 149 | 207 |
| 평균 | | 136.8451 | 200.0000 | 175.7102 | 200.0000 | 161.1818 | 200.0000 | 154.6781 | 185.7906 |
| 중위수 | | 166.0000 | 200.0000 | 200.0000 | 200.0000 | 200.0000 | 200.0000 | 200.0000 | 200.0000 |
| 표준편차 | | 68.19764 | .00000 | 106.12332 | .00000 | 55.80335 | .00000 | 55.39920 | 36.48415 |
| 백분위수 | 25 | 70.0000 | 200.0000 | 152.0000 | 200.0000 | 121.2500 | 200.0000 | 100.0000 | 200.0000 |
|  | 50 | 166.0000 | 200.0000 | 200.0000 | 200.0000 | 200.0000 | 200.0000 | 200.0000 | 200.0000 |
|  | 75 | 200.0000 | 200.0000 | 200.0000 | 200.0000 | 200.0000 | 200.0000 | 200.0000 | 200.0000 |

*Abbrevations

Macintosh laryngoscope – mac,dl

Glidescope –gvl

I-gel- igel
