# Supplementary material for: Protection afforded by respirators when performing endotracheal intubation using a direct laryngoscope, GlideScope®, and i-gel® device: A randomized trial
Source: PLoS One. 2018 Apr 19;13(4):e0195745. doi: 10.1371/journal.pone.0195745 (PMC5909605; doi:10.1371/journal.pone.0195745)
Supplement: S6 File — (DOCX) [file pone.0195745.s006.docx]

GET

FILE='D:\OneDrive\HYER\Mask Fit Test\[HYER]마스크 실험 결과 분석\[최종 통계] -4- 기관 삽관 장비의 종류\후두경 종류 마스크 통계 20171209\mask fit test_mask '+

'laryngotypes-20171219.sav'.

DATASET NAME 데이터집합1 WINDOW=FRONT.

GET

FILE='D:\OneDrive\HYER\Mask Fit Test\[HYER]마스크 실험 결과 분석\[최종 통계] -4- 기관 삽관 장비의 종류\후두경 종류 마스크 통계 20171209\mask_ '+

'laryngo_friedman.sav'.

DATASET NAME 데이터집합2 WINDOW=FRONT.

NPAR TESTS

/FRIEDMAN=dl_cup dl_fold gvl_cup gvl_fold igel_cup igel_fold

/MISSING LISTWISE.

**비모수 검정**

| **노트** | | |
| --- | --- | --- |
| 작성된 출력결과 | | 16-DEC-2017 14:56:34 |
| 주석 | |  |
| 입력 | 데이터 | D:\OneDrive\HYER\Mask Fit Test\[HYER]마스크 실험 결과 분석\[최종 통계] -4- 기관 삽관 장비의 종류\후두경 종류 마스크 통계 20171209\mask_ laryngo_friedman.sav |
|  | 활성 데이터 집합 | 데이터집합2 |
|  | 필터 | <지정않음> |
|  | 가중 | <지정않음> |
|  | 파일분할 | <지정않음> |
|  | 작업 데이터 파일의 행 수 | 441 |
| 결측값 처리 | 결측값 정의 | 사용자 정의 결측값은 누락된 데이터로 처리됩니다. |
|  | 사용 케이스 | 모든 검정에 대한 통계량은 사용된 변수에 대한 결측 데이터가 없는 케이스를 기준으로 결정됩니다. |
| 구문 | | NPAR TESTS  /FRIEDMAN=dl_cup dl_fold gvl_cup gvl_fold igel_cup igel_fold  /MISSING LISTWISE. |
| 자원 | 프로세서 시간 | 00:00:00.00 |
|  | 경과 시간 | 00:00:00.00 |
|  | 허용된 케이스의 수^a^ | 71493 |
| a. 작업 공간 메모리의 가용성을 기준으로. | | |

[데이터집합2] D:\OneDrive\HYER\Mask Fit Test\[HYER]마스크 실험 결과 분석\[최종 통계] -4- 기관 삽관 장비의 종류\후두경 종류 마스크 통계 20171209\mask_ laryngo_friedman.sav

**Friedman 검정**

| **순위** | |
| --- | --- |
|  | 평균순위 |
| dl_cup | 2.12 |
| dl_fold | 4.27 |
| gvl_cup | 3.09 |
| gvl_fold | 4.27 |
| igel_cup | 2.99 |
| igel_fold | 4.27 |

| **검정 통계량^a^** | |
| --- | --- |
| N | 209 |
| 카이제곱 | 448.286 |
| 자유도 | 5 |
| 근사 유의확률 | .000 |
| a. Friedman 검정 | |

NPAR TESTS

/WILCOXON=dl_cup dl_cup gvl_cup dl_fold dl_fold gvl_fold WITH gvl_cup igel_cup igel_cup gvl_fold igel_fold igel_fold (PAIRED)

/MISSING ANALYSIS.

**비모수 검정**

| **노트** | | |
| --- | --- | --- |
| 작성된 출력결과 | | 16-DEC-2017 14:59:14 |
| 주석 | |  |
| 입력 | 데이터 | D:\OneDrive\HYER\Mask Fit Test\[HYER]마스크 실험 결과 분석\[최종 통계] -4- 기관 삽관 장비의 종류\후두경 종류 마스크 통계 20171209\mask_ laryngo_friedman.sav |
|  | 활성 데이터 집합 | 데이터집합2 |
|  | 필터 | <지정않음> |
|  | 가중 | <지정않음> |
|  | 파일분할 | <지정않음> |
|  | 작업 데이터 파일의 행 수 | 441 |
| 결측값 처리 | 결측값 정의 | 사용자 정의 결측값은 누락된 데이터로 처리됩니다. |
|  | 사용 케이스 | 각 검정에 대한 통계량은 해당 검정에 사용된 변수에 대한 유효 데이터를 포함하는 모든 케이스를 기준으로 결정됩니다. |
| 구문 | | NPAR TESTS  /WILCOXON=dl_cup dl_cup gvl_cup dl_fold dl_fold gvl_fold WITH gvl_cup igel_cup igel_cup gvl_fold igel_fold igel_fold (PAIRED)  /MISSING ANALYSIS. |
| 자원 | 프로세서 시간 | 00:00:00.00 |
|  | 경과 시간 | 00:00:00.01 |
|  | 허용된 케이스의 수^a^ | 71493 |
| a. 작업 공간 메모리의 가용성을 기준으로. | | |

[데이터집합2] D:\OneDrive\HYER\Mask Fit Test\[HYER]마스크 실험 결과 분석\[최종 통계] -4- 기관 삽관 장비의 종류\후두경 종류 마스크 통계 20171209\mask_ laryngo_friedman.sav

**Wilcoxon 부호순위 검정**

| **순위** | | | | |
| --- | --- | --- | --- | --- |
|  | | N | 평균순위 | 순위합 |
| gvl_cup - dl_cup | 음의 순위 | 74^a^ | 105.55 | 7810.50 |
|  | 양의 순위 | 190^b^ | 143.00 | 27169.50 |
|  | 동률 | 119^c^ |  |  |
|  | 합계 | 383 |  |  |
| igel_cup - dl_cup | 음의 순위 | 45^d^ | 76.12 | 3425.50 |
|  | 양의 순위 | 120^e^ | 85.58 | 10269.50 |
|  | 동률 | 49^f^ |  |  |
|  | 합계 | 214 |  |  |
| igel_cup - gvl_cup | 음의 순위 | 78^g^ | 65.07 | 5075.50 |
|  | 양의 순위 | 59^h^ | 74.19 | 4377.50 |
|  | 동률 | 77^i^ |  |  |
|  | 합계 | 214 |  |  |
| gvl_fold - dl_fold | 음의 순위 | 0^j^ | .00 | .00 |
|  | 양의 순위 | 0^k^ | .00 | .00 |
|  | 동률 | 396^l^ |  |  |
|  | 합계 | 396 |  |  |
| igel_fold - dl_fold | 음의 순위 | 0^m^ | .00 | .00 |
|  | 양의 순위 | 0^n^ | .00 | .00 |
|  | 동률 | 209^o^ |  |  |
|  | 합계 | 209 |  |  |
| igel_fold - gvl_fold | 음의 순위 | 0^p^ | .00 | .00 |
|  | 양의 순위 | 0^q^ | .00 | .00 |
|  | 동률 | 209^r^ |  |  |
|  | 합계 | 209 |  |  |
| a. gvl_cup < dl_cup | | | | |
| b. gvl_cup > dl_cup | | | | |
| c. gvl_cup = dl_cup | | | | |
| d. igel_cup < dl_cup | | | | |
| e. igel_cup > dl_cup | | | | |
| f. igel_cup = dl_cup | | | | |
| g. igel_cup < gvl_cup | | | | |
| h. igel_cup > gvl_cup | | | | |
| i. igel_cup = gvl_cup | | | | |
| j. gvl_fold < dl_fold | | | | |
| k. gvl_fold > dl_fold | | | | |
| l. gvl_fold = dl_fold | | | | |
| m. igel_fold < dl_fold | | | | |
| n. igel_fold > dl_fold | | | | |
| o. igel_fold = dl_fold | | | | |
| p. igel_fold < gvl_fold | | | | |
| q. igel_fold > gvl_fold | | | | |
| r. igel_fold = gvl_fold | | | | |

| **검정 통계량^a^** | | | | | | |
| --- | --- | --- | --- | --- | --- | --- |
|  | gvl_cup - dl_cup | igel_cup - dl_cup | igel_cup - gvl_cup | gvl_fold - dl_fold | igel_fold - dl_fold | igel_fold - gvl_fold |
| Z | -7.795^b^ | -5.568^b^ | -.750^c^ | .000^d^ | .000^d^ | .000^d^ |
| 근사 유의확률(양측) | .000 | .000 | .453 | 1.000 | 1.000 | 1.000 |
| a. Wilcoxon 부호순위 검정 | | | | | | |
| b. 음의 순위를 기준으로. | | | | | | |
| c. 양의 순위를 기준으로. | | | | | | |
| d. 음의 순위의 합계는 양의 순위의 합계와 같습니다. | | | | | | |
