# Supplementary material for: Protection afforded by respirators when performing endotracheal intubation using a direct laryngoscope, GlideScope®, and i-gel® device: A randomized trial
Source: PLoS One. 2018 Apr 19;13(4):e0195745. doi: 10.1371/journal.pone.0195745 (PMC5909605; doi:10.1371/journal.pone.0195745)
Supplement: S7 File — (DOCX) [file pone.0195745.s007.docx]

**데이터 탐색**

GET

FILE='C:\Users\user\Desktop\전 후 비교 mask fit test_mask laryngotypes-20171219 -.sav'.

DATASET NAME 데이터집합1 WINDOW=FRONT.

SORT CASES BY larygo_type mask_type.

SPLIT FILE SEPARATE BY larygo_type mask_type.

EXAMINE VARIABLES=mask_fittest

/PLOT BOXPLOT NPPLOT

/COMPARE GROUPS

/STATISTICS DESCRIPTIVES

/CINTERVAL 95

/MISSING LISTWISE

/NOTOTAL.

| **노트** | | |
| --- | --- | --- |
| 작성된 출력결과 | | 09-DEC-2017 21:54:10 |
| 주석 | |  |
| 입력 | 데이터 | C:\Users\user\Desktop\전 후 비교 mask fit test_mask laryngotypes-20171219 -.sav |
|  | 활성 데이터 집합 | 데이터집합1 |
|  | 필터 | <지정않음> |
|  | 가중 | <지정않음> |
|  | 파일분할 | larygo_type, 1.cup,2fold,3valve |
|  | 작업 데이터 파일의 행 수 | 2597 |
| 결측값 처리 | 결측값 정의 | 종속변수에 대한 사용자 정의 결측값은 누락된 데이터로 처리됩니다. |
|  | 사용 케이스 | 통계량은 사용된 종속변수나 요인에 대한 결측값이 없는 케이스를 기준으로 결정됩 니다. |
| 구문 | | EXAMINE VARIABLES=mask_fittest  /PLOT BOXPLOT NPPLOT  /COMPARE GROUPS  /STATISTICS DESCRIPTIVES  /CINTERVAL 95  /MISSING LISTWISE  /NOTOTAL. |
| 사용된 자원 | 프로세서 시간 | 00:00:04.03 |
|  | 경과 시간 | 00:00:03.21 |

[데이터집합1] C:\Users\user\Desktop\전 후 비교 mask fit test_mask laryngotypes-20171219 -.sav

**larygo_type = ., 1.cup,2fold,3valve = cup type**

| **케이스 처리 요약^a^** | | | | | | |
| --- | --- | --- | --- | --- | --- | --- |
|  | 케이스 | | | | | |
|  | 유효 | | 결측 | | 전체 | |
|  | N | 퍼센트 | N | 퍼센트 | N | 퍼센트 |
| mask_fittest | 292 | 100.0% | 0 | 0.0% | 292 | 100.0% |
| a. larygo_type = ., 1.cup,2fold,3valve = cup type | | | | | | |

| **기술통계^a^** | | | | |
| --- | --- | --- | --- | --- |
|  | | | 통계량 | 표준오차 |
| mask_fittest | 평균 | | 154.6781 | 3.24199 |
|  | 평균의 95% 신뢰구간 | 하한 | 148.2974 |  |
|  |  | 상한 | 161.0588 |  |
|  | 5% 절삭평균 | | 157.9140 |  |
|  | 중위수 | | 200.0000 |  |
|  | 분산 | | 3069.071 |  |
|  | 표준편차 | | 55.39920 |  |
|  | 최소값 | | 45.00 |  |
|  | 최대값 | | 200.00 |  |
|  | 범위 | | 155.00 |  |
|  | 사분위수 범위 | | 100.00 |  |
|  | 왜도 | | -.653 | .143 |
|  | 첨도 | | -1.252 | .284 |
| a. larygo_type = ., 1.cup,2fold,3valve = cup type | | | | |

| **정규성 검정^a^** | | | | | | |
| --- | --- | --- | --- | --- | --- | --- |
|  | Kolmogorov-Smirnov^b^ | | | Shapiro-Wilk | | |
|  | 통계량 | 자유도 | 유의확률 | 통계량 | 자유도 | 유의확률 |
| mask_fittest | .317 | 292 | .000 | .758 | 292 | .000 |
| a. larygo_type = ., 1.cup,2fold,3valve = cup type | | | | | | |
| b. Lilliefors 유의확률 수정 | | | | | | |

**mask_fittest**

**larygo_type = ., 1.cup,2fold,3valve = fold type**

| **케이스 처리 요약^a^** | | | | | | |
| --- | --- | --- | --- | --- | --- | --- |
|  | 케이스 | | | | | |
|  | 유효 | | 결측 | | 전체 | |
|  | N | 퍼센트 | N | 퍼센트 | N | 퍼센트 |
| mask_fittest | 234 | 100.0% | 0 | 0.0% | 234 | 100.0% |
| a. larygo_type = ., 1.cup,2fold,3valve = fold type | | | | | | |

| **기술통계^a^** | | | | |
| --- | --- | --- | --- | --- |
|  | | | 통계량 | 표준오차 |
| mask_fittest | 평균 | | 185.7906 | 2.38504 |
|  | 평균의 95% 신뢰구간 | 하한 | 181.0916 |  |
|  |  | 상한 | 190.4896 |  |
|  | 5% 절삭평균 | | 191.3675 |  |
|  | 중위수 | | 200.0000 |  |
|  | 분산 | | 1331.093 |  |
|  | 표준편차 | | 36.48415 |  |
|  | 최소값 | | 20.00 |  |
|  | 최대값 | | 200.00 |  |
|  | 범위 | | 180.00 |  |
|  | 사분위수 범위 | | .00 |  |
|  | 왜도 | | -2.489 | .159 |
|  | 첨도 | | 4.915 | .317 |
| a. larygo_type = ., 1.cup,2fold,3valve = fold type | | | | |

| **정규성 검정^a^** | | | | | | |
| --- | --- | --- | --- | --- | --- | --- |
|  | Kolmogorov-Smirnov^b^ | | | Shapiro-Wilk | | |
|  | 통계량 | 자유도 | 유의확률 | 통계량 | 자유도 | 유의확률 |
| mask_fittest | .489 | 234 | .000 | .441 | 234 | .000 |
| a. larygo_type = ., 1.cup,2fold,3valve = fold type | | | | | | |
| b. Lilliefors 유의확률 수정 | | | | | | |

**mask_fittest**

**larygo_type = 1.00, 1.cup,2fold,3valve = cup type**

| **케이스 처리 요약^a^** | | | | | | |
| --- | --- | --- | --- | --- | --- | --- |
|  | 케이스 | | | | | |
|  | 유효 | | 결측 | | 전체 | |
|  | N | 퍼센트 | N | 퍼센트 | N | 퍼센트 |
| mask_fittest | 441 | 100.0% | 0 | 0.0% | 441 | 100.0% |
| a. larygo_type = 1.00, 1.cup,2fold,3valve = cup type | | | | | | |

| **기술통계^a^** | | | | |
| --- | --- | --- | --- | --- |
|  | | | 통계량 | 표준오차 |
| mask_fittest | 평균 | | 136.8451 | 3.24751 |
|  | 평균의 95% 신뢰구간 | 하한 | 130.4626 |  |
|  |  | 상한 | 143.2277 |  |
|  | 5% 절삭평균 | | 140.0322 |  |
|  | 중위수 | | 166.0000 |  |
|  | 분산 | | 4650.919 |  |
|  | 표준편차 | | 68.19764 |  |
|  | 최소값 | | .00 |  |
|  | 최대값 | | 200.00 |  |
|  | 범위 | | 200.00 |  |
|  | 사분위수 범위 | | 130.00 |  |
|  | 왜도 | | -.479 | .116 |
|  | 첨도 | | -1.397 | .232 |
| a. larygo_type = 1.00, 1.cup,2fold,3valve = cup type | | | | |

| **정규성 검정^a^** | | | | | | |
| --- | --- | --- | --- | --- | --- | --- |
|  | Kolmogorov-Smirnov^b^ | | | Shapiro-Wilk | | |
|  | 통계량 | 자유도 | 유의확률 | 통계량 | 자유도 | 유의확률 |
| mask_fittest | .263 | 441 | .000 | .808 | 441 | .000 |
| a. larygo_type = 1.00, 1.cup,2fold,3valve = cup type | | | | | | |
| b. Lilliefors 유의확률 수정 | | | | | | |

**mask_fittest**

**larygo_type = 1.00, 1.cup,2fold,3valve = fold type**

| **케이스 처리 요약^a^** | | | | | | |
| --- | --- | --- | --- | --- | --- | --- |
|  | 케이스 | | | | | |
|  | 유효 | | 결측 | | 전체 | |
|  | N | 퍼센트 | N | 퍼센트 | N | 퍼센트 |
| mask_fittest | 396 | 100.0% | 0 | 0.0% | 396 | 100.0% |
| a. larygo_type = 1.00, 1.cup,2fold,3valve = fold type | | | | | | |

| **기술통계^a^** | | | | |
| --- | --- | --- | --- | --- |
|  | | | 통계량 | 표준오차 |
| mask_fittest | 평균 | | 189.2614 | 5.02295 |
|  | 평균의 95% 신뢰구간 | 하한 | 179.3863 |  |
|  |  | 상한 | 199.1364 |  |
|  | 5% 절삭평균 | | 191.7890 |  |
|  | 중위수 | | 200.0000 |  |
|  | 분산 | | 9991.109 |  |
|  | 표준편차 | | 99.95554 |  |
|  | 최소값 | | 9.60 |  |
|  | 최대값 | | 2000.00 |  |
|  | 범위 | | 1990.40 |  |
|  | 사분위수 범위 | | .00 |  |
|  | 왜도 | | 14.913 | .123 |
|  | 첨도 | | 274.112 | .245 |
| a. larygo_type = 1.00, 1.cup,2fold,3valve = fold type | | | | |

| **정규성 검정^a^** | | | | | | |
| --- | --- | --- | --- | --- | --- | --- |
|  | Kolmogorov-Smirnov^b^ | | | Shapiro-Wilk | | |
|  | 통계량 | 자유도 | 유의확률 | 통계량 | 자유도 | 유의확률 |
| mask_fittest | .455 | 396 | .000 | .170 | 396 | .000 |
| a. larygo_type = 1.00, 1.cup,2fold,3valve = fold type | | | | | | |
| b. Lilliefors 유의확률 수정 | | | | | | |

**mask_fittest**

**larygo_type = 2.00, 1.cup,2fold,3valve = cup type**

| **케이스 처리 요약^a^** | | | | | | |
| --- | --- | --- | --- | --- | --- | --- |
|  | 케이스 | | | | | |
|  | 유효 | | 결측 | | 전체 | |
|  | N | 퍼센트 | N | 퍼센트 | N | 퍼센트 |
| mask_fittest | 383 | 100.0% | 0 | 0.0% | 383 | 100.0% |
| a. larygo_type = 2.00, 1.cup,2fold,3valve = cup type | | | | | | |

| **기술통계^a^** | | | | |
| --- | --- | --- | --- | --- |
|  | | | 통계량 | 표준오차 |
| mask_fittest | 평균 | | 175.7102 | 5.42265 |
|  | 평균의 95% 신뢰구간 | 하한 | 165.0482 |  |
|  |  | 상한 | 186.3722 |  |
|  | 5% 절삭평균 | | 176.8697 |  |
|  | 중위수 | | 200.0000 |  |
|  | 분산 | | 11262.159 |  |
|  | 표준편차 | | 106.12332 |  |
|  | 최소값 | | 10.00 |  |
|  | 최대값 | | 2002.00 |  |
|  | 범위 | | 1992.00 |  |
|  | 사분위수 범위 | | 48.00 |  |
|  | 왜도 | | 13.218 | .125 |
|  | 첨도 | | 230.449 | .249 |
| a. larygo_type = 2.00, 1.cup,2fold,3valve = cup type | | | | |

| **정규성 검정^a^** | | | | | | |
| --- | --- | --- | --- | --- | --- | --- |
|  | Kolmogorov-Smirnov^b^ | | | Shapiro-Wilk | | |
|  | 통계량 | 자유도 | 유의확률 | 통계량 | 자유도 | 유의확률 |
| mask_fittest | .407 | 383 | .000 | .268 | 383 | .000 |
| a. larygo_type = 2.00, 1.cup,2fold,3valve = cup type | | | | | | |
| b. Lilliefors 유의확률 수정 | | | | | | |

**mask_fittest**

**larygo_type = 2.00, 1.cup,2fold,3valve = fold type**

| **케이스 처리 요약^a^** | | | | | | |
| --- | --- | --- | --- | --- | --- | --- |
|  | 케이스 | | | | | |
|  | 유효 | | 결측 | | 전체 | |
|  | N | 퍼센트 | N | 퍼센트 | N | 퍼센트 |
| mask_fittest | 428 | 100.0% | 0 | 0.0% | 428 | 100.0% |
| a. larygo_type = 2.00, 1.cup,2fold,3valve = fold type | | | | | | |

| **기술통계^a^** | | | | |
| --- | --- | --- | --- | --- |
|  | | | 통계량 | 표준오차 |
| mask_fittest | 평균 | | 193.7453 | 1.26677 |
|  | 평균의 95% 신뢰구간 | 하한 | 191.2554 |  |
|  |  | 상한 | 196.2352 |  |
|  | 5% 절삭평균 | | 199.0099 |  |
|  | 중위수 | | 200.0000 |  |
|  | 분산 | | 686.813 |  |
|  | 표준편차 | | 26.20712 |  |
|  | 최소값 | | 20.00 |  |
|  | 최대값 | | 200.00 |  |
|  | 범위 | | 180.00 |  |
|  | 사분위수 범위 | | .00 |  |
|  | 왜도 | | -5.026 | .118 |
|  | 첨도 | | 26.154 | .235 |
| a. larygo_type = 2.00, 1.cup,2fold,3valve = fold type | | | | |

| **정규성 검정^a^** | | | | | | |
| --- | --- | --- | --- | --- | --- | --- |
|  | Kolmogorov-Smirnov^b^ | | | Shapiro-Wilk | | |
|  | 통계량 | 자유도 | 유의확률 | 통계량 | 자유도 | 유의확률 |
| mask_fittest | .508 | 428 | .000 | .256 | 428 | .000 |
| a. larygo_type = 2.00, 1.cup,2fold,3valve = fold type | | | | | | |
| b. Lilliefors 유의확률 수정 | | | | | | |

**mask_fittest**

**larygo_type = 3.00, 1.cup,2fold,3valve = cup type**

| **케이스 처리 요약^a^** | | | | | | |
| --- | --- | --- | --- | --- | --- | --- |
|  | 케이스 | | | | | |
|  | 유효 | | 결측 | | 전체 | |
|  | N | 퍼센트 | N | 퍼센트 | N | 퍼센트 |
| mask_fittest | 214 | 100.0% | 0 | 0.0% | 214 | 100.0% |
| a. larygo_type = 3.00, 1.cup,2fold,3valve = cup type | | | | | | |

| **기술통계^a^** | | | | |
| --- | --- | --- | --- | --- |
|  | | | 통계량 | 표준오차 |
| mask_fittest | 평균 | | 161.1818 | 3.81464 |
|  | 평균의 95% 신뢰구간 | 하한 | 153.6625 |  |
|  |  | 상한 | 168.7011 |  |
|  | 5% 절삭평균 | | 166.1563 |  |
|  | 중위수 | | 200.0000 |  |
|  | 분산 | | 3114.014 |  |
|  | 표준편차 | | 55.80335 |  |
|  | 최소값 | | 6.80 |  |
|  | 최대값 | | 200.00 |  |
|  | 범위 | | 193.20 |  |
|  | 사분위수 범위 | | 78.75 |  |
|  | 왜도 | | -1.135 | .166 |
|  | 첨도 | | -.139 | .331 |
| a. larygo_type = 3.00, 1.cup,2fold,3valve = cup type | | | | |

| **정규성 검정^a^** | | | | | | |
| --- | --- | --- | --- | --- | --- | --- |
|  | Kolmogorov-Smirnov^b^ | | | Shapiro-Wilk | | |
|  | 통계량 | 자유도 | 유의확률 | 통계량 | 자유도 | 유의확률 |
| mask_fittest | .331 | 214 | .000 | .721 | 214 | .000 |
| a. larygo_type = 3.00, 1.cup,2fold,3valve = cup type | | | | | | |
| b. Lilliefors 유의확률 수정 | | | | | | |

**mask_fittest**

**larygo_type = 3.00, 1.cup,2fold,3valve = fold type**

| **케이스 처리 요약^a^** | | | | | | |
| --- | --- | --- | --- | --- | --- | --- |
|  | 케이스 | | | | | |
|  | 유효 | | 결측 | | 전체 | |
|  | N | 퍼센트 | N | 퍼센트 | N | 퍼센트 |
| mask_fittest | 209 | 100.0% | 0 | 0.0% | 209 | 100.0% |
| a. larygo_type = 3.00, 1.cup,2fold,3valve = fold type | | | | | | |

| **기술통계^a^** | | | | |
| --- | --- | --- | --- | --- |
|  | | | 통계량 | 표준오차 |
| mask_fittest | 평균 | | 198.3923 | .61935 |
|  | 평균의 95% 신뢰구간 | 하한 | 197.1713 |  |
|  |  | 상한 | 199.6134 |  |
|  | 5% 절삭평균 | | 200.0000 |  |
|  | 중위수 | | 200.0000 |  |
|  | 분산 | | 80.172 |  |
|  | 표준편차 | | 8.95390 |  |
|  | 최소값 | | 123.00 |  |
|  | 최대값 | | 200.00 |  |
|  | 범위 | | 77.00 |  |
|  | 사분위수 범위 | | .00 |  |
|  | 왜도 | | -6.250 | .168 |
|  | 첨도 | | 40.665 | .335 |
| a. larygo_type = 3.00, 1.cup,2fold,3valve = fold type | | | | |

| **정규성 검정^a^** | | | | | | |
| --- | --- | --- | --- | --- | --- | --- |
|  | Kolmogorov-Smirnov^b^ | | | Shapiro-Wilk | | |
|  | 통계량 | 자유도 | 유의확률 | 통계량 | 자유도 | 유의확률 |
| mask_fittest | .523 | 209 | .000 | .176 | 209 | .000 |
| a. larygo_type = 3.00, 1.cup,2fold,3valve = fold type | | | | | | |
| b. Lilliefors 유의확률 수정 | | | | | | |

**mask_fittest**
