# Supplementary material for: Protection afforded by respirators when performing endotracheal intubation using a direct laryngoscope, GlideScope®, and i-gel® device: A randomized trial
Source: PLoS One. 2018 Apr 19;13(4):e0195745. doi: 10.1371/journal.pone.0195745 (PMC5909605; doi:10.1371/journal.pone.0195745)
Supplement: S8 File — (DOCX) [file pone.0195745.s008.docx]

SAVE OUTFILE='C:\Users\user\Desktop\mask_ laryngo_friedman.sav'

/COMPRESSED.

NPAR TESTS

/FRIEDMAN=dl_cup dl_fold gvl_cup gvl_fold igel_cup igel_fold

/MISSING LISTWISE.

**비모수 검정**

| **노트** | | |
| --- | --- | --- |
| 작성된 출력결과 | | 09-DEC-2017 21:29:01 |
| 주석 | |  |
| 입력 | 데이터 | C:\Users\user\Desktop\mask_ laryngo_friedman.sav |
|  | 활성 데이터 집합 | 데이터집합0 |
|  | 필터 | <지정않음> |
|  | 가중 | <지정않음> |
|  | 파일분할 | <지정않음> |
|  | 작업 데이터 파일의 행 수 | 441 |
| 결측값 처리 | 결측값 정의 | 사용자 정의 결측값은 누락된 데이터로 처리됩니다. |
|  | 사용 케이스 | 모든 검정에 대한 통계량은 사용된 변수에 대한 결측 데이터가 없는 케이스를 기준으로 결정됩니다. |
| 구문 | | NPAR TESTS  /FRIEDMAN=dl_cup dl_fold gvl_cup gvl_fold igel_cup igel_fold  /MISSING LISTWISE. |
| 자원 | 프로세서 시간 | 00:00:00.00 |
|  | 경과 시간 | 00:00:00.00 |
|  | 허용된 케이스의 수^a^ | 71493 |
| a. 작업 공간 메모리의 가용성을 기준으로. | | |

[데이터집합0] C:\Users\user\Desktop\mask_ laryngo_friedman.sav

**Friedman 검정**

| **순위** | |
| --- | --- |
|  | 평균순위 |
| dl_cup | 2.12 |
| dl_fold | 4.27 |
| gvl_cup | 3.09 |
| gvl_fold | 4.27 |
| igel_cup | 2.99 |
| igel_fold | 4.27 |

| **검정 통계량^a^** | |
| --- | --- |
| N | 209 |
| 카이제곱 | 448.286 |
| 자유도 | 5 |
| 근사 유의확률 | .000 |
| a. Friedman 검정 | |

NPAR TESTS

/WILCOXON=dl_cup gvl_cup igel_cup WITH dl_fold gvl_fold igel_fold (PAIRED)

/MISSING ANALYSIS.

**비모수 검정**

| **노트** | | |
| --- | --- | --- |
| 작성된 출력결과 | | 09-DEC-2017 21:30:45 |
| 주석 | |  |
| 입력 | 데이터 | C:\Users\user\Desktop\mask_ laryngo_friedman.sav |
|  | 활성 데이터 집합 | 데이터집합0 |
|  | 필터 | <지정않음> |
|  | 가중 | <지정않음> |
|  | 파일분할 | <지정않음> |
|  | 작업 데이터 파일의 행 수 | 441 |
| 결측값 처리 | 결측값 정의 | 사용자 정의 결측값은 누락된 데이터로 처리됩니다. |
|  | 사용 케이스 | 각 검정에 대한 통계량은 해당 검정에 사용된 변수에 대한 유효 데이터를 포함하는 모든 케이스를 기준으로 결정됩니다. |
| 구문 | | NPAR TESTS  /WILCOXON=dl_cup gvl_cup igel_cup WITH dl_fold gvl_fold igel_fold (PAIRED)  /MISSING ANALYSIS. |
| 자원 | 프로세서 시간 | 00:00:00.00 |
|  | 경과 시간 | 00:00:00.00 |
|  | 허용된 케이스의 수^a^ | 71493 |
| a. 작업 공간 메모리의 가용성을 기준으로. | | |

[데이터집합0] C:\Users\user\Desktop\mask_ laryngo_friedman.sav

**Wilcoxon 부호순위 검정**

| **순위** | | | | |
| --- | --- | --- | --- | --- |
|  | | N | 평균순위 | 순위합 |
| dl_fold - dl_cup | 음의 순위 | 0^a^ | .00 | .00 |
|  | 양의 순위 | 226^b^ | 113.50 | 25651.00 |
|  | 동률 | 170^c^ |  |  |
|  | 합계 | 396 |  |  |
| gvl_fold - gvl_cup | 음의 순위 | 1^d^ | 128.00 | 128.00 |
|  | 양의 순위 | 127^e^ | 64.00 | 8128.00 |
|  | 동률 | 255^f^ |  |  |
|  | 합계 | 383 |  |  |
| igel_fold - igel_cup | 음의 순위 | 0^g^ | .00 | .00 |
|  | 양의 순위 | 90^h^ | 45.50 | 4095.00 |
|  | 동률 | 119^i^ |  |  |
|  | 합계 | 209 |  |  |
| a. dl_fold < dl_cup | | | | |
| b. dl_fold > dl_cup | | | | |
| c. dl_fold = dl_cup | | | | |
| d. gvl_fold < gvl_cup | | | | |
| e. gvl_fold > gvl_cup | | | | |
| f. gvl_fold = gvl_cup | | | | |
| g. igel_fold < igel_cup | | | | |
| h. igel_fold > igel_cup | | | | |
| i. igel_fold = igel_cup | | | | |

| **검정 통계량^a^** | | | |
| --- | --- | --- | --- |
|  | dl_fold - dl_cup | gvl_fold - gvl_cup | igel_fold - igel_cup |
| Z | -13.034^b^ | -9.513^b^ | -8.239^b^ |
| 근사 유의확률(양측) | .000 | .000 | .000 |
| a. Wilcoxon 부호순위 검정 | | | |
| b. 음의 순위를 기준으로. | | | |

사용권 갱신 기간이 지났습니다. 새 사용권을 곧 설치하지 않으면 이 제품의 작동이 중지됩니다.
